# Essential function of alveolin *Pf*IMC1g in the *Plasmodium falciparum* asexual blood stage

**DOI:** 10.1128/mbio.01507-23

**Published:** 2023-09-15

**Authors:** Ana Karla Cepeda Diaz, Rachel M. Rudlaff, Madeline Farringer, Jeffrey D. Dvorin

**Affiliations:** 1 Division of Infectious Diseases, Boston Children’s Hospital, Boston, Massachusetts, USA; 2 Biological and Biomedical Sciences, Harvard Medical School, Boston, Massachusetts, USA; 3 Biological Sciences in Public Health, Harvard T.H. Chan School of Public Health, Boston, Massachusetts, USA; 4 Department of Pediatrics, Harvard Medical School, Boston, Massachusetts, USA; George Washington University, Washington, DC, USA

**Keywords:** malaria, *Plasmodium*, cell biology, cytoskeleton, alveolin

## Abstract

The cytoskeleton of *Plasmodium* parasites is essential for replication, motility, and infectivity. *Plasmodium falciparum* leverages a family of cytoskeletal proteins known as alveolins to meet these diverse needs. The functional role of individual alveolins in *Plasmodium* blood stages, however, remains unexplored. Here, we demonstrate that the alveolin *Pf*IMC1g (PF3D7_0525800) is essential for *P. falciparum* asexual replication. Unlike alveolins studied in mosquito stages, *Pf*IMC1g does not play an important role in determining cell shape. *Pf*IMC1g-deficient parasites exhibit only minor defects during segmentation, with most merozoites being indistinguishable from wild type by super-resolution fluorescence microscopy, ultrastructure expansion microscopy, and electron microscopy. In the current study, we demonstrate that the absence of *Pf*IMC1g leads to parasite death shortly after merozoite internalization into red blood cells (RBCs). *Pf*IMC1g-deficient parasites egress and enter new RBCs but fail to develop into rings and die. We hypothesize that the primary role of *Pf*IMC1g is to maintain structural integrity, protecting parasites from incurring damage during the process of internalization. Along with the dispensability of *Pf*IMC1g for merozoite cell shape, we report new findings about the architecture of *Plasmodium* alveolins including the localization of *Pf*IMC1e and 1f to the basal complex.

**Importance:**

Infection by the *Plasmodium falciparum* parasite is responsible for the most severe form of human malaria. The asexual blood stage of the parasite, which occurs inside human red blood cells, is responsible for the symptoms of malaria and is the target of most antimalarial drugs. *Plasmodium* spp. rely on their highly divergent cytoskeletal structures to scaffold their cell division, sustain the mechanical stress of invasion, and survive in both the human bloodstream and the mosquito. We investigate the function of a class of divergent intermediate filament-like proteins called alveolins in the clinically important blood stage. The functional role of individual alveolins in *Plasmodium* remains poorly understood due to pleiotropic effects of gene knockouts and redundancy among alveolins. We evaluate the localization and essentiality of the four asexual-stage alveolins and find that *Pf*IMC1g and *Pf*IMC1c are essential. Furthermore, we demonstrate that *Pf*IMC1g is critical for survival of the parasite post-invasion.

## INTRODUCTION

*Plasmodium* parasites continue to cause significant global morbidity and mortality, with over 240 million cases of malaria and 600,000 deaths in 2020 (1). To complete their lifecycle, *Plasmodium* parasites must go from mosquito to human and back, infecting multiple tissues in the process. Consequently, the ability of *Plasmodium* spp. to invade new cells is both essential for the spread of malaria and directly responsible for its symptoms. The molecular structures that enable parasite survival during and following the mechanical stress of invasion, however, remain poorly understood.

Unlike animal cytoskeletons, alveolate cytoskeletal systems are built around a distinctive cortical structure called the pellicle, which in *Plasmodium* and other apicomplexans comprises the plasma membrane and an organelle known as the inner membrane complex (IMC) (2). The IMC is an endomembrane structure with associated proteins that serves a cytoskeleton-like function in conjunction with the meshwork of proteins that lies directly interior to it, the subpellicular network (SPN) (3). As a whole, the pellicle partitions cytoplasmic contents during segmentation, dictates cell shape, and provides essential structural support (4 – 6). Studying these divergent structures in *Plasmodium falciparum*, the causative agent of the deadliest form of human malaria, promises to reveal much-needed novel drug targets. However, the short-lived nature of *P. falciparum* merozoites, the parasite’s infectious blood stage, has made it challenging to measure their biophysical properties or link these properties to any specific protein or invasion phenotype.

It has been suggested that the subpellicular microtubules (MTs), which line the IMC of *Plasmodium* mosquito stages, provide the structural integrity needed for invasion (7). However, *P. falciparum* merozoites lack the array of microtubules present in other invasive stages. Instead, merozoites have a single spine of two to three microtubules which runs from the parasite’s apical ring toward its basal complex (BC) (8). Merozoites must derive the mechanical properties which allow them to survive invasion from elsewhere, perhaps because they have distinct cytoskeletal requirements incompatible with a microtubule corset.

Besides tubulin, the other major component of the SPN is a family of intermediate filament (IF)-like proteins called alveolins. These proteins form filaments 8–10 nm in diameter, organizing into a regularly (~32 nm) spaced mesh in *Plasmodium* sporozoites (9) and the related parasite *Toxoplasma gondii* (2). Alveolins are evolutionarily distinct from metazoan intermediate filaments, despite sharing many of their biophysical attributes. Their complete absence outside the kingdom Alveolata, and sequence divergence from IFs, makes alveolins a promising drug target (10). So far, 13 alveolins have been identified in *Plasmodium* and named *Pf*IMC1a–1m. They differ greatly in expression pattern and size, but all have one or more valine- and proline-rich “alveolin repeat” (IMCp) domains (11).

Studies in *Plasmodium berghei*, which is infectious in rodents, have demonstrated that some alveolins have stage-specific functions and expression patterns. For example, *Pb*IMC1a, 1b, and 1 h knockouts lead to defects in the shape, tensile strength, and infectivity of *P. berghei* sporozoites and ookinetes (5, 12, 13). To our knowledge, there has not yet been a comparable analysis in *P. falciparum* or characterization of any *Plasmodium* alveolin in the asexual blood stages. Furthermore, it remains unclear whether the pleiotropic effects of alveolin knockouts indicate multifunctionality of these proteins or a common mechanism to these phenotypes. Thus, the molecular and stage-specific roles of the alveolins remain unknown. In merozoites, *Pf*IMC1c/PF3D7_1003600, *Pf*IMC1e/PF3D7_0304100, *Pf*IMC1f/PF3D7_1351700, and *Pf*IMC1g/PF3D7_0525800 are the main alveolin components of the SPN as predicted by transcriptomics (14). Previous studies have observed *Pf*IMC1g in schizonts, where its localization is consistent with that of the subpellicular network (15). Furthermore, *Pf*IMC1g was predicted to be essential for survival and replication in the blood stages by the piggyBac transposon insertion mutagenesis screen (16). In this work, we examined the role of *Pf*IMC1g in merozoite cell shape and red blood cell (RBC) invasion to see if this protein contributes to the cytoskeletal properties that allow merozoites to survive the mechanical stress of invasion in RBCs.

## RESULTS

### *Pf*IMC1g is essential for *P. falciparum* asexual replication

**Fig 1:**
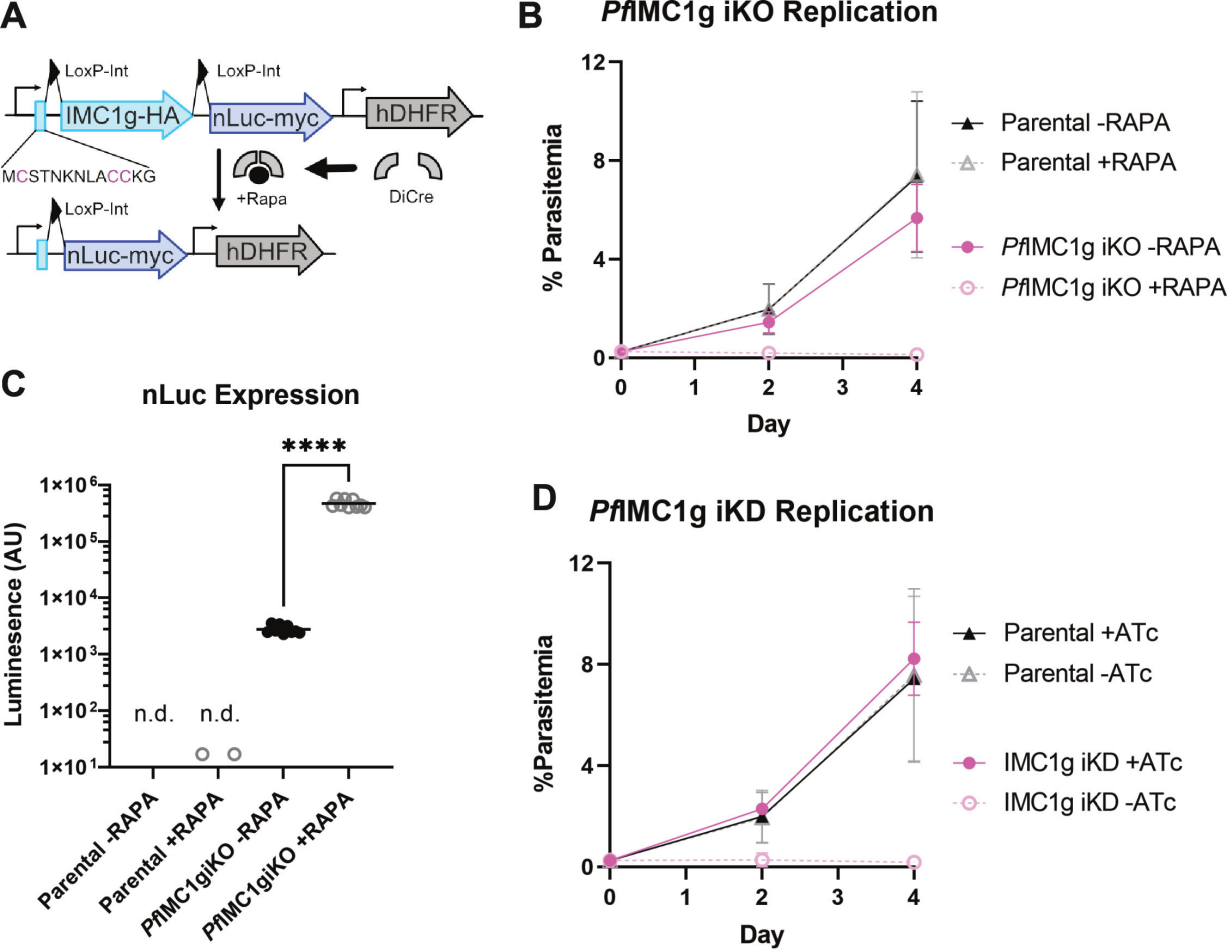
*Pf*IMC1g is essential for asexual replication. (**A**) Diagram of *Pf*IMC1g iKO construct. Predicted palmitoylation sites in pink. LoxP sites are inserted in synthetic introns. *Pf*IMC1g is excised upon addition of RAPA, yielding a fusion protein of the *Pf*IMC1g N-term with nLuc. (**B**) Replication of *Pf*IMC1g wild type (WT) (−RAPA) and KO (+RAPA) parasites, in pink, and their parental line, in black, as measured by flow cytometry. Graph shows mean ± SD of three biological replicates. (**C**) Nanoluciferase assay of *Pf*IMC1g iKO line without induction of excision (−RAPA) and with induction (+RAPA) compared to its parental (3D7 DiCre). Each point represents a technical replicate, *n* = 9 (three biological replicates with three technical replicates each). n.d. indicates at least one point is not shown because its value was not detected. ****, *P* < 0.0001 (unpaired *t*-test). (**D**) Replication of *Pf*IMC1g WT (+ATc) and KD (−ATc) parasites, in pink, and their parental line, in black, as measured by flow cytometry. Graph shows mean ± SD of three biological replicates. Atc, anhydrotetracycline.

To confirm the essentiality of *Pf*IMC1g and further investigate its role in asexual blood-stage replication, we generated inducible knockdown (iKD) and inducible knockout (iKO) lines of *Pf*IMC1g as well as a primary antibody against it (Fig. 1; Fig. S1). We generated a *Pf*IMC1g iKO line by placing two synthetic introns with LoxP sites, previously developed for use in *Plasmodium* (17), into the *Pf*IMC1g locus. The first intron was placed 20aa downstream from the *Pf*IMC1g start codon (Fig. 1A), and the second was placed after the stop codon of an HA_x2_ tag at the C-terminal end of *Pf*IMC1g. This second intron is followed by nanoluciferase (nLuc) to report successful *Pf*IMC1g excision (Fig. 1C). This line also contains a dimerizable Cre (DiCre) recombinase controlled by rapamycin (RAPA), such that addition of rapamycin induces excision of *Pf*IMC1g (18).

We also generated an iKD line using the TetR-DOZI system, which was previously adapted for use in *Plasmodium*, to conditionally deplete the mRNA of *Pf*IMC1g using the TetR ligand anhydrotetracycline (ATc) (19, 20). In brief, removal of ATc results in the sequestration of *Pf*IMC1g mRNA into stress granules and prevents its translation. We generated both an untagged *Pf*IMC1g iKD line and a line with a C-terminal spaghetti monster V5 tag (smV5) (21). While parasites of the untagged line maintained in ATc had a similar replication rate to its parental line, parasites tagged with smV5 had a lower parasite multiplication rate (Fig. S2). This indicates that the C-terminal domain of *P*IMC1g may be important for its function, and placement of the bulky smV5 tag may interfere with this putative function.

Both our iKD and iKO systems allowed us to achieve levels of *Pf*IMC1g undetectable by western blot in the absence of ATc and presence of rapamycin, respectively (Fig. S1). Parasite replication was completely abolished in *Pf*IMC1g-deficient conditions in both lines, as measured by flow cytometry (Fig. 1B and D). No infected RBCs were detected up to 4 days post-knockdown, and no rings were observed by Field’s (Hemacolor) stain after the first cycle. Despite lacking a large tag, our iKO line showed a lower parasite multiplication rate than its parental line and had above-background nLuc expression in the absence of rapamycin (Fig. 1C). Given these observations, we could not be sure whether any phenotype observed under iKO conditions relative to control was solely a product of *Pf*IMC1g deficiency. To address this limitation, we performed phenotypic characterization of PfIMC1g using both the iKO and untagged iKD lines in parallel to account for possible artifactual effects of the iKO.

### IMC1g-deficient parasites have a segmentation defect, leading to a small reduction in total number of viable daughter cells

**Fig 2:**
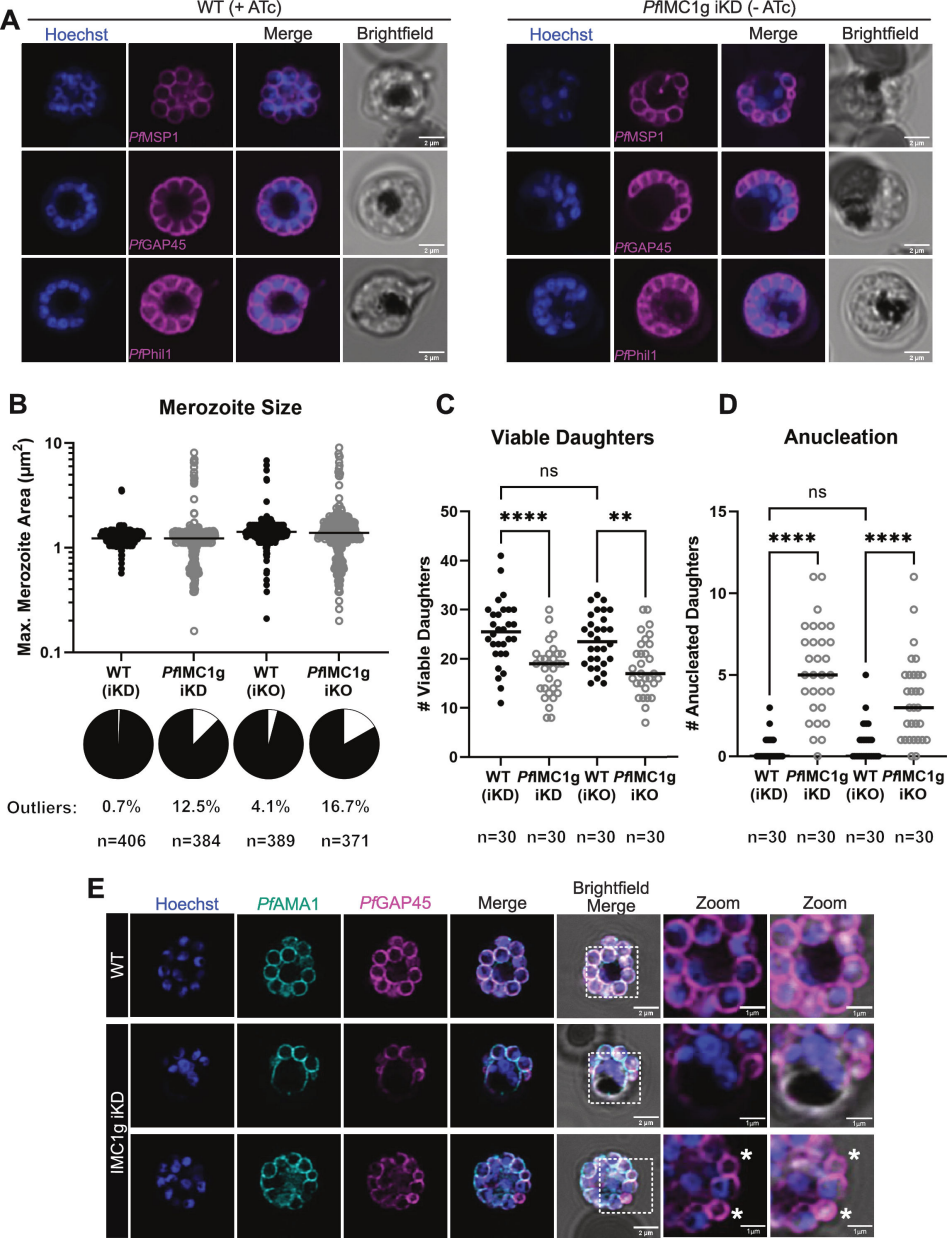
*Pf*IMC1g-deficient parasites have a segmentation defect, leading to a slight reduction in the number of viable daughter cells produced. (**A**) Airyscan super-resolution images of merozoite plasma membrane (*Pf*MSP1) and inner membrane complex (*Pf*GAP45 and *Pf*PhIl1) markers in schizonts under *Pf*IMC1g WT (+ATc) and KD (−ATc) conditions. Scale bars = 2 µm. (**B**) Mean and distribution of merozoite size reported as area of maximum cross-section. Each point represents a daughter cell, columns show pooled data from three biological replicates. Pie charts show proportion of outliers (>3 SD from WT mean) in white. (**C**) Mean and distribution of number of viable daughter cells produced and (D) anucleated daughter cells produced. Columns in both graphs represent data from 30 schizonts across three biological replicates, where each point represents data from one schizont. ****, *P* < 0.0001; **, *P* < 0.01 (ordinary one-way ANOVA, Dunnett multiplicity adjusted *P*-values). (**E**) Airyscan super-resolution images of translocated microneme protein *Pf*AMA1 and inner membrane complex marker *Pf*GAP45 in schizonts under *Pf*IMC1g WT (+ATc) and KD (−ATc) conditions. Scale bars = 2 µm. Zoomed images highlight aberrant residual body and anucleated cells (*) in KD parasites. Scale bars = 1 µm.

To characterize daughter cell shape and size during schizogony, we used immunofluorescence assays (IFAs) for *Pf*GAP45 (22) and *Pf*PhIL1 (23) as markers of IMC integrity and biogenesis and *Pf*MSP1 (24) as a plasma membrane marker. While WT (+ATc) cells were uniform in size and exhibited the expected coordination of segmentation across all proteins visualized, *Pf*IMC1g-deficient (−ATc) cells were heterogeneous and produced daughter cells of inconsistent size (Fig. 2A). To quantify this phenotype, we imaged WT (+ATc) and *Pf*IMC1g-deficient (−ATc) post-egress parasites arrested with the protease inhibitor E64 for 2–3 h prior to harvest. This inhibitor allows for normal daughter cell maturation but prevents parasites from rupturing the RBC plasma membrane once segmentation is complete (25). To ensure that the parasites imaged were fully segmented and the observed differences were not a product of a developmental delay, we imaged only schizonts which had a single hemozoin crystal and showed translocation of microneme protein *Pf*AMA1 to the parasite plasma membrane. From this data set, we quantified the area of the largest cross-section of each daughter cell as a measure of merozoite size. To our surprise, the mean size of merozoites was not different between WT and iKD (Fig. 2B; mean daughter size WT [+ATc] = 1.26 µm^2^; iKD [−ATc] = 1.31 µm^2^; analysis of variance [ANOVA] with Welch’s correction *P* > 0.99). Rather, there was a difference in variance (Brown-Forsythe test *P* < 0.0001). On average, 12.50% of daughter cells produced by *Pf*IMC1g-deficient (−ATc) parasites are of aberrant size (>3 SD from the WT [+ATc] mean). Thus, most daughters produced by *Pf*IMC1g-deficient parasites are of the same size as those produced by WT parasites.

We also quantified the number of anucleation events (daughters lacking a nucleus) per schizont. There is a high incidence of anucleation in *Pf*IMC1g-deficient parasites (Fig. 2D; mean anucleation events WT [+ATc] = 0.40; KD [−ATc] = 5.30 anucleated daughters/schizont) and a few schizonts have, in addition, an enlarged residual body with >2 nuclei (Fig. 2E, middle row). However, in quantifying the number of viable daughter cells (daughters of size within 3 SD of the WT mean, with a single nucleus, and with translocated AMA1) we noticed that WT parasites only make, on average, ~7 more viable daughters than *Pf*IMC1g-deficient parasites (Fig. 2C; mean WT [+ATc] = 25.33; mean KD [−ATc] = 17.80 daughters/schizont, ANOVA with Welch’s correction *P* < 0.0001). While this could explain a small reduction in replication factor, this difference in the number of viable daughter cells is not enough to account for the complete lethality of *Pf*IMC1g depletion (Fig. 1B and D).

To assess whether residual *Pf*IMC1g could explain the small degree of this segmentation defect, we stained iKD (−ATc) parasites against *Pf*IMC1g by IFA. Residual *Pf*IMC1g distribution did not correlate with daughter size, confirming that this minor segmentation defect is not due to residual *Pf*IMC1g (Fig. S1b). Furthermore, the merozoite size distribution observed in our iKO (+RAPA) conditions was indistinguishable from that of the KD (−ATc) (Fig. 2; mean daughter size KD [−ATc] = 1.31 ± 0.92 µm^2^; KO [+RAPA] = 1.51 ± 1 µm^2^; Brown-Forsythe test to compare variance *P* = 0.12). In addition, there was no significant difference in the number of anucleation events or number of viable daughters produced (Fig. 2C and D; mean anucleation events KO [+RAPA] = 3.47 anucleated daughters/schizont; mean viable daughters KO [+RAPA] = 18.33 daughters/schizont) Thus, complete loss of *Pf*IMC1g does not lead to a more severe segmentation defect than depletion by iKD.

Thus, even with complete KO of *Pf*IMC1g, we fail to detect a segmentation phenotype that could account for the complete lethality of *Pf*IMC1g deficiency. This could be because aberrations caused by *Pf*IMC1g deficiency lie at a finer ultrastructural level. Thus, we looked in more detail at potential disruption to the ultrastructure and cytoskeleton of *Pf*IMC1g-deficient parasites.

### Loss of *Pf*IMC1g does not affect the tubulin cytoskeleton

It has been observed that disruption of alveolin network-associated proteins in *T. gondii* disrupts the parasite’s conoid and subpellicular microtubules (26). While a similar effect was not observed in *P. berghei* sporozoites (5), the organizing principles and architecture of the merozoite cytoskeleton remain largely unexplored. So, we stained parasites with antibodies against tubulin to determine whether the disruption to the alveolin meshwork caused by *Pf*IMC1g deficiency led to corresponding disruptions in the subpellicular microtubules, beyond what could be explained by the difference in cell size. While tubulin appeared highly heterogeneous in length by IFA, this approach did not provide sufficient resolution to answer this question (Fig. S3). Therefore, we used ultrastructure expansion microscopy (U-ExM) (27) to look at tubulin in the context of schizont ultrastructure.

**Fig 3:**
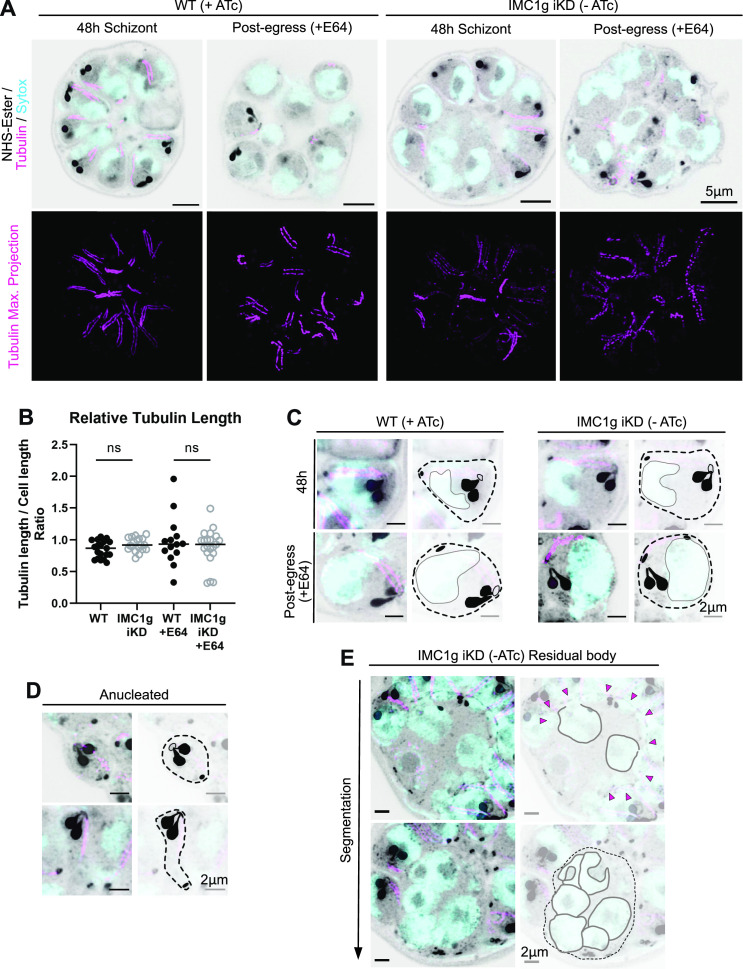
*Pf*IMC1g-deficient parasites lack ultrastructural abnormalities by U-ExM. (**A**) Airyscan super-resolution images of expanded schizonts under *Pf*IMC1g WT (+ATc) and KD (−ATc) conditions. Parasites were stained with DNA-dye SYTOX Deep Red, amine-reactive dye NHS-ester, and anti-tubulin primary antibody. Single slice (top), maximum projection (bottom). Scale bars = 5 µm. (**B**) Mean and distribution of relative tubulin length of each daughter cell in an individual schizont. Relative length calculated as tubulin length (linear distance) divided by the linear distance from APR to BC. Each column represents an individual schizont; the two columns on the right represent post-egress schizonts (+E64). ns, not significant (*P* > 0.05, ordinary one-way ANOVA). (C) Maximum *z*-projection of individual merozoites in WT and *Pf*IMC1g-deficient parasites. Scale bars = 2 µm. (**D**) Maximum *z*-projection of anucleated daughter cells and (E) enlarged residual body in *Pf*IMC1g-deficient parasites. Scale bars = 2 µm. Diagrams show rhoptries, APR, and BC (solid black), cell boundaries (dotted line), contracting basal complex (magenta arrowheads), and nuclei (gray outlines).

We stained expanded parasites with an amine-reactive NHS-ester conjugated to a fluorophore, in addition to using a primary antibody against tubulin (Fig. 3). NHS-ester allows us to visualize protein-rich structures including the rhoptries, the basal complex, and the apical polar ring (APR). Using these images, we measured the length of each subpellicular microtubule bundle and compared it to cell length, measured as distance from the APR to the basal complex (Fig. 3B). There was no significant difference in the ratio of tubulin length to APR-BC distance between WT and *Pf*IMC1g-deficient parasites (mean length ratio WT [+ATc] = 0.87; KD [−ATc] = 0.93 µm, one-way ANOVA *P* = 0.15). Though we observed a larger variance in E64-arrested parasites, this is likely due to the disorganized, random orientation of E64 arrested parasites, which makes it more challenging to accurately pair basal complex and apical polar rings belonging to the same parasite in both WT (+ATc) and *Pf*IMC1g-deficient (−ATc) conditions. Furthermore, the correlation between cell length and tubulin length in WT (+ATc) parasites (Pearson *r* = 0.90, *P* < 0.0001) and KD parasites (−ATc) (Pearson *r* = 0.87, *P* < 0.0001) was not significantly different (*z*-test on Fisher *z*-transformed correlation coefficients *P* = 0.20; Fig. S3B). This indicates that the length of tubulin relative to cell size is not affected by *Pf*IMC1g KD.

### Loss of *Pf*IMC1g does not affect parasite ultrastructure

U-ExM combined with NHS-ester staining additionally allowed us to obtain 3D reconstructions of WT (+ATc) and *Pf*IMC1g-deficient (−ATc) schizonts at a resolution allowing individual parasite organelles to be distinguished, to better assess potential cell shape and ultrastructural defects. In these images, *Pf*IMC1g-deficient parasites have APR, basal complex, and rhoptries comparable to those of WT cells regardless of cell size (Fig. 3C and D). This suggests that *Pf*IMC1g deficiency does not affect apical organelle biogenesis in schizonts. In parasites with segmentation defects, the residual body contains four to six nuclei on average, in addition to additional protein-dense material we were not able to conclusively identify by NHS-ester staining (Fig. 3E). Anucleated daughters appear either small and rounded or flexible and elongated, suggesting *Pf*IMC1g deficiency could cause an increase in deformability (Fig. 3D). However, it is possible that this increase in deformability is shared by nucleated *Pf*IMC1g-deficient daughters, and only becomes apparent by U-ExM in the absence of a nucleus. Despite these morphological abnormalities, *Pf*IMC1g-deficient anucleated daughters have rhoptries, APRs, and basal complexes indistinguishable from those of WT parasites (Fig. 3C through E).

**Fig 4:**
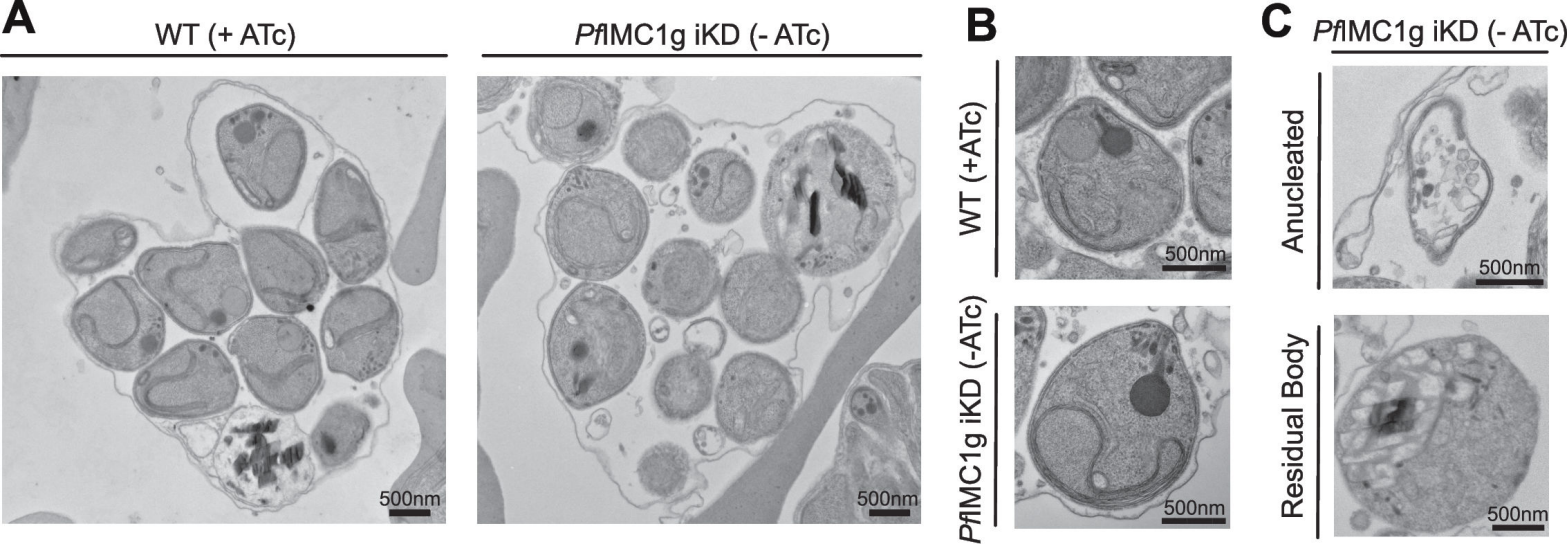
*Pf*IMC1g-deficient parasites lack ultrastructural abnormalities by TEM. (**A**) Transmission electron micrographs of schizonts and (B) individual daughter cells cultured with ATc (WT) and without ATc (KD). (**C**) Anucleated daughter cell (top) and enlarged residual body (bottom) from *Pf*IMC1g-deficient parasites. Scale bars = 500 nm.

We supplemented these studies by examining parasite ultrastructure using standard transmission electron microscopy (TEM). No apparent differences in parasite and organelle morphology were observed between E64-arrested WT (+ATc) and *Pf*IMC1g-deficient (−ATc) parasites (Fig. 4). Chemical fixation and dehydration used for TEM can affect cell size and morphology. Thus, the cell morphology observed in these TEM studies may not reflect the natural state of the parasite. The effects of this sample preparation, however, should be consistent between WT (+ATc) and KD (−ATc) parasites, and any aberrations which arise disproportionally in the KD could indicate a larger biological phenomenon we have been otherwise unable to measure. Despite lacking ultrastructural differences, KD parasites appeared rounder than WT parasites by TEM. We quantified and confirmed this difference in roundness using particle analysis in ImageJ (Fig. S3C; mean roundness score WT [+ATc ]= 0.83, KD [−ATc ]= 0.89, *P* < 0.0001). This suggests that *Pf*IMC1g-deficient parasites may have stiffness or permeability different from that of WT parasites, leading to different reactions to the TEM preparation. This fits with observations in the mosquito stages of *Plasmodium* that mutating or knocking out certain alveolins leads to decreased tensile strength of ookinetes in a hypoosmotic shock assay (28). WT and *Pf*IMC1g-deficient parasites were indistinguishable from each other beyond this difference in post-fixation roundness, the enlarged residual body, and the presence of anucleated daughters in the iKD.

### IMC1g-deficient parasites egress normally after segmentation and have functional apical organelles

**Fig 5:**
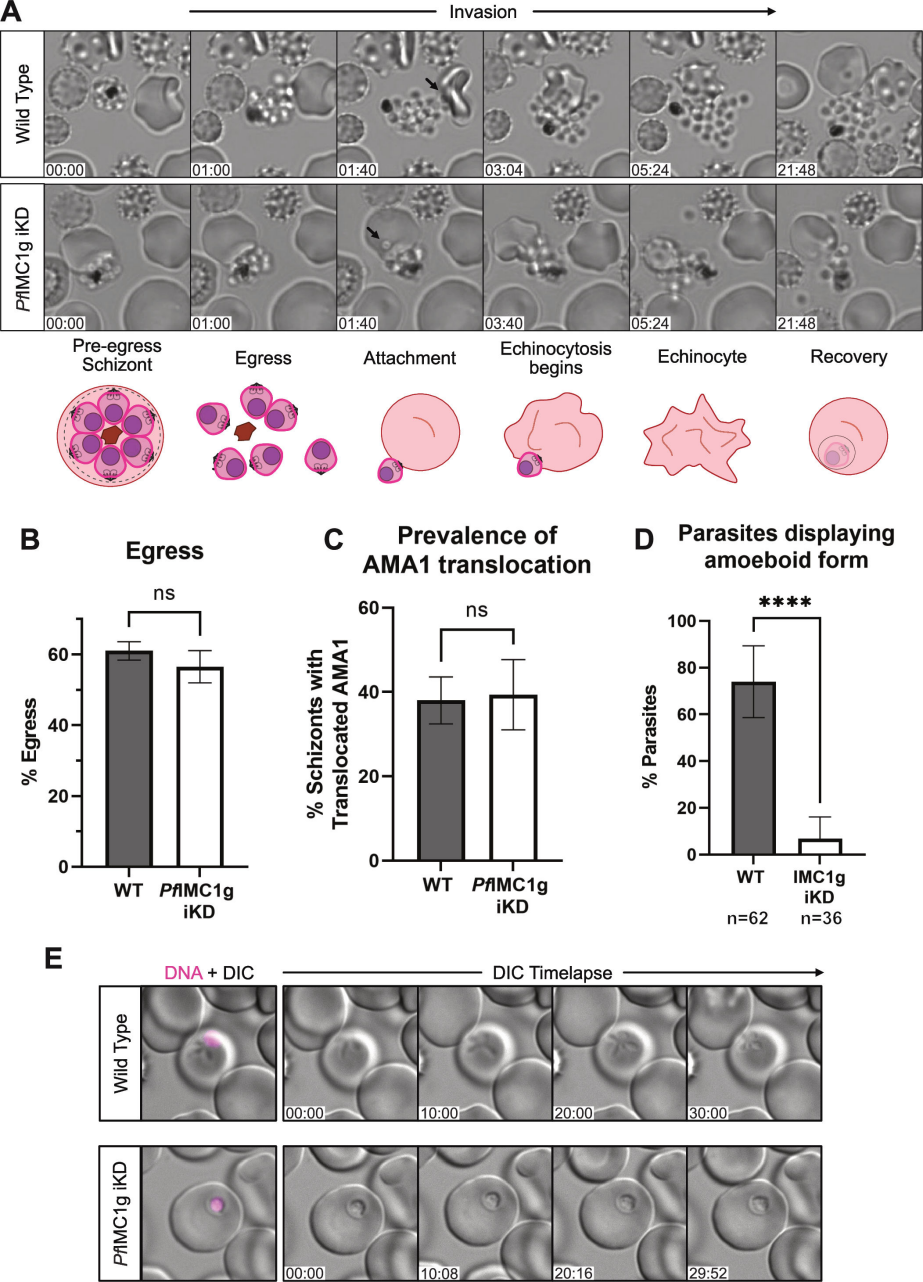
*Pf*IMC1g-deficient parasites egress normally after segmentation but do not survive internalization. (**A**) Live cell microscopy stills of WT (+ATc) and *Pf*IMC1g KD (−ATc) schizonts undergoing egress and invasion of new RBCs. First frame is set at 1 min before the first sign of egress. Timestamp MM:SS. Magenta arrows indicate attached merozoites. (**B**) Parasite egress as quantified by flow cytometry. % Egress = 100 * (1 − (schizontemia at 6 hpi/ schizontemia at 0 hpi)), where schizontemia is the proportion of RBCs ≥ 3N and hpi is hours post-invasion. Graph shows mean ± SD of four biological replicates. ns, not significant (*P* > 0.05, unpaired *t*-test). (C) Quantification of microneme function by IFA. The graph shows the proportion of schizonts which have translocated *Pf*AMA1 to the plasma membrane after being arrested with E64. Graph shows mean ± SD of three biological replicates. ns, not significant (*P* > 0.05, unpaired *t*-test). (D) Percentage of RBCs showing amoeboid parasites at 3 h after C1 release. Graph shows mean ± SD of three biological replicates. ****, *P* < 0.0001 (unpaired *t*-test). (**E**) Representative images of time-lapse differential interference contrast (DIC) imaging of RBCs positive for parasite material. Hoechst (magenta) was used as a DNA stain.

To further assess whether parasite apical organelles, which are key for invasion, retain their function in the absence of *Pf*IMC1g, we quantified the translocation of microneme protein *Pf*AMA1 (29, 30). We harvested WT (+ATc) and *Pf*IMC1g-deficient (−ATc) schizonts which had been arrested with E64 for 4 h and stained for *Pf*AMA1 by IFA. We quantified the prevalence of *Pf*AMA1 translocation by dividing the number of schizonts where *Pf*AMA1 had translocated from the apical end to the cell plasma membrane in one or more daughters by the total number of schizonts which stained positive for *Pf*AMA1. We observed no difference in *Pf*AMA1 translocation at a population level (Fig. 5C; mean translocation WT [+ATc] = 38%; KD [−ATc] = 39%).

We used time-lapse microscopy to observe whether *Pf*IMC1g deficiency affects parasite egress and RBC invasion. To our surprise, these videos showed that at least some *Pf*IMC1g-deficient daughter cells were able to complete invasion and become internalized by the RBC. *Pf*IMC1g-deficient merozoite egress, attachment, and induction of echinocytosis is indistinguishable from that of WT parasites by brightfield microscopy (Fig. 5A). To confirm these observations, we quantified egress and re-invasion by flow cytometry, tracking DNA content of RBCs using a DNA stain. WT (+ATc) and KD (−ATc) parasites egressed at the same rates (Fig. 5B), but only WT parasites produced newly infected RBCs (Fig. 1D). These data confirm that our time-lapse microscopy observations reflect population-level trends. Since we did not observe any defect in *Pf*AMA1 translocation, internalization, or triggering of echinocytosis, we concluded that *Pf*IMC1g deficiency is unlikely to cause a defect in apical organelle function.

### *Pf*IMC1g deficiency affects daughter cell survival after internalization

Given the surprising results of our time-lapse microscopy, we examined the fate of tightly synchronized parasites 3 h and 20 h post-invasion by Field’s stain. This revealed that at 3 hpi, the percentage of RBCs staining positive for parasite material was only 27% lower in *Pf*IMC1g-deficient (−ATc) parasites than their wild-type (+ATc) control (Fig. S4A). This difference is consistent with the loss in viable parasites produced by the minor segmentation defect described above (~30% decrease in viable daughter cells). The close match between the difference between WT and *Pf*IMC1g-deficient conditions in the two experiments suggests that parasite attachment, reorientation, and actinomyosin-dependent motility in normal-sized merozoites remain sufficient to meet the parasite’s invasion needs.

To assess whether these internalized parasites had normal morphology, we categorized 100 parasites at each time point into one of three morphologies: ring, early trophozoite, and unknown/aberrant (Fig. S4B). Briefly, parasites categorized into rings and trophozoites have traits specific to those stages, while parasites of unknown/aberrant morphology have ambiguous traits such that they cannot be discerned as either dead or *bona fide* rings (see detailed categorization criteria in Table S1). At 3 hpi, RBCs in wild-type conditions have a substantial number of rings (WT [+ATc] 3 hpi rings = 25%, unknown/aberrant = 75%; Fig. S4C). In contrast, rings are rarely observed in the *Pf*IMC1g-deficient condition (*Pf*IMC1g KD [−ATc] 3 hpi rings = 6%, unknown/aberrant = 94%). This confirms the observation we made in our time-lapse microscopy that *Pf*IMC1g-deficient parasites seem to successfully enter new RBCs but suggests that subsequent steps do not progress normally.

At 3 hpi, heparin was added to the cultures to prevent further invasion, and the cultures were checked again 17 h later after washing away extracellular parasite debris. At 20 hpi, the percentage of RBCs that stained positive for parasite material in WT conditions matched that recorded at 3 h, with most parasites having attained a ring or early trophozoite morphology [WT (+ATc) 20 hpi rings = 35%, early trophozoite = 25%, unknown/aberrant = 40%; Fig. S4C]. However, few RBCs stained positive for parasite material in the *Pf*IMC1g-deficient (−ATc) condition (mean loss in parasitemia = 91.15%). This suggests *Pf*IMC1g-deficient daughter cells die inside RBCs during or shortly after internalization. It is likely that RBCs with dead parasite material inside them eventually lyse.

### Death of IMC1g-deficient daughter cells is a catastrophic event that occurs within 3 h of invasion

To investigate whether the aberrant parasite morphology we observed by Field’s stain was due to parasite death, we imaged live invaded RBCs at 3 hpi by differential interference contrast (DIC) microscopy (Fig. 5; Video S1). Invaded RBCs were identified using a membrane-permeable DNA dye and parasites were imaged every 30 s for 30 min to determine whether they displayed amoeboid forms, the first morphological marker of ring development (31). We observed parasite amoeboid forms in 74% of DNA-positive RBCs in the WT (+ATc) condition and in 7% of DNA-positive RBCs in the IMC1g iKD (−ATc) condition (Fig. 5D). Most internalized IMC1g iKD parasites appeared small and rounded, suggesting the aberrant morphologies we observed by Field’s stain were not a product of fixation and indicating that parasites are likely already dead at this point (Fig. 5E). In our live cell microscopy, we routinely notice merozoites in both PfIMC1g-sufficient and PfIMC1g-deficient conditions which fail to invade but still attain amoeboid form outside the RBC (Fig. S5A). This suggests that *Pf*IMC1g-deficient parasites are not *ipso facto* incapable of adopting the amoeboid form but rather that they lose this ability in the process of invasion.

**Fig 6:**
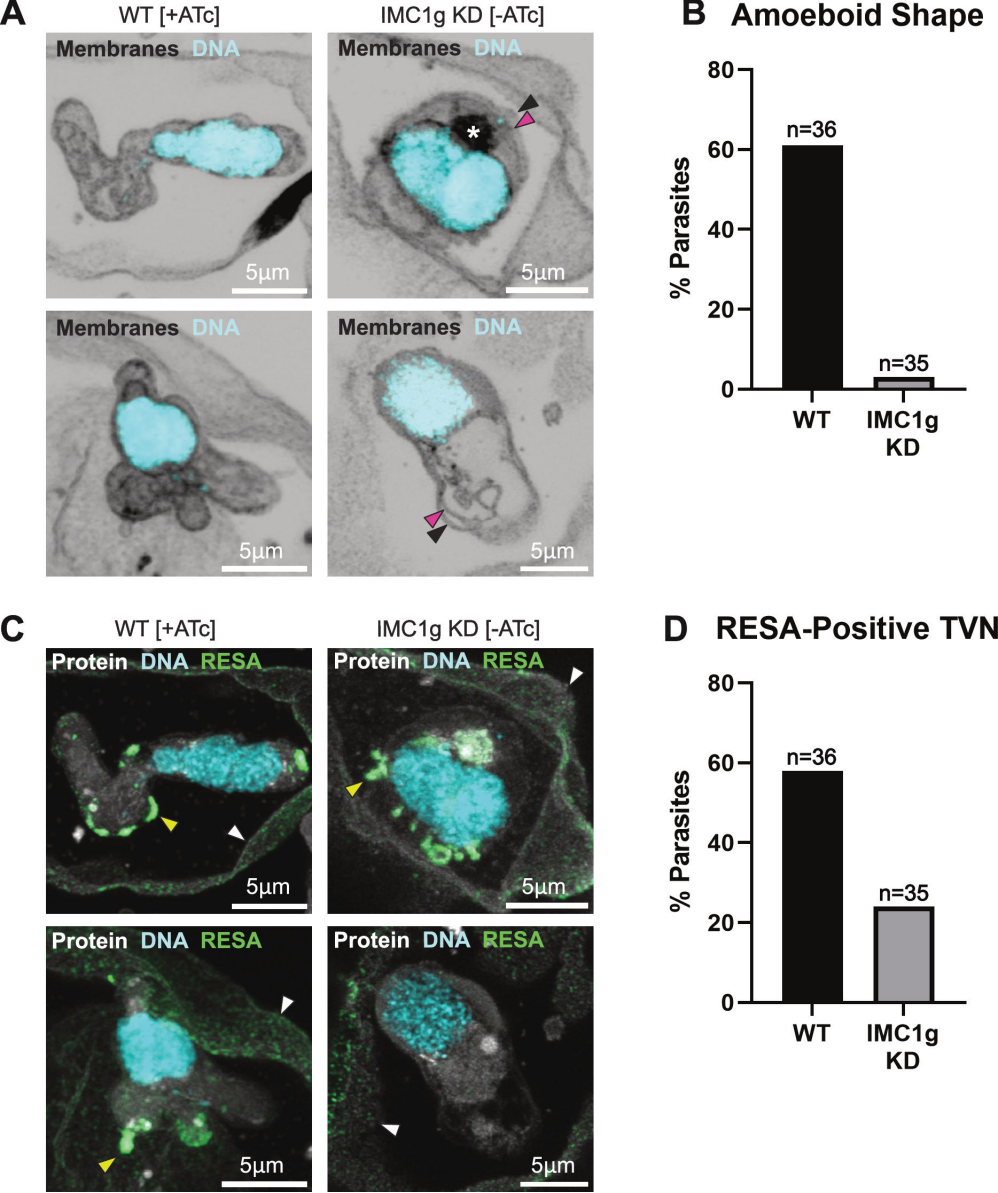
*Pf*IMC1g-deficient parasites complete invasion up to RESA discharge and parasitophorous vacuole formation. (**A**) U-ExM images of parasites harvested at 3 hpi under *Pf*IMC1g WT (+ATc) (left) and KD (−ATc) (right) conditions. Parasites were stained with DNA-dye SYTOX Deep Red (cyan) and BODIPY-Trc (membranes). Black arrowhead, parasitophorous vacuole membrane; magenta arrowhead, parasite plasma membrane; white asterisk, high-intensity staining of membranes (potentially, the disassembling IMC). (**B**) Percentage of imaged RBCs showing amoeboid parasites. Graph shows pooled data from two biological replicates. (**C**) U-ExM images of parasites harvested at 3 hpi under *Pf*IMC1g WT (+ATc) (left) and KD (−ATc) (right) conditions. Parasites stained with DNA-dye SYTOX Deep Red, amine-reactive AF-405-conjugated NHS-ester, and primary antibody against RESA. White arrowhead, RBC plasma membrane; yellow arrowhead, RESA-positive tubulovesicular network (TVN). (**D**) Percentage of imaged RBCs showing RESA-positive TVN. Graph shows pooled data from two biological replicates. Scale bars = 5 µm.

To characterize the nature of the damage incurred by parasites during invasion, we looked for ultrastructural abnormalities in internalized parasites at 3 hpi using expansion microscopy (Fig. 6). A DNA dye and BODIPY-TRc, a dye-conjugated fatty acid, were used to examine the integrity of the nucleus and other membranous structures (Fig. 6A). Parasites were also stained with a fluorophore-conjugated NHS-ester as a marker of protein density to visualize the host-cell cytoplasm and protein-dense parasite features (Fig. 6C). Finally, to further pinpoint time of death, parasites were stained against ring-infected erythrocyte surface antigen (RESA). This dense granule protein is released inside the RBC on entry and trafficked to the RBC membrane (32).

As observed by DIC, most WT (+ATc) parasites showed amoeboid form (61%), while morphology suggestive of amoeboid form was only observed in 3% of IMC1g-deficient (−ATc) parasites (Fig. 6B). Besides the absence of amoeboid forms, no obvious cause of death was observed in IMC1g-deficient parasites, which showed no abnormalities in their nuclear membrane, plasma membrane, or parasitophorous vacuole membrane (Fig. 6A).

Nearly all RBCs which had internalized parasites showed RESA staining at their plasma membranes regardless of IMC1g status (RESA-positive RBCs WT [+ATc] = 97%; KD [−ATc] = 97%). This suggests that all imaged parasites carry out initial discharge of their dense granules and begin the process of RBC remodeling. While 21 of 36 (58%) WT (+ATc) parasites imaged across two biological replicates had RESA staining in their tubulovesicular network (TVN), this was only observed in 8 of 34 (24%) imaged IMC1g-deficient (−ATc) parasites (Fig. 6D). These structures have been observed to appear almost immediately (<12 min) after invasion (33). Interestingly, all KD (−ATc) parasites that did have RESA-positive TVNs also had membranous foci which were rarely observed in WT (+ATc) parasites. Membranous foci similar to these have been previously described as disassembling IMC which has detached from the plasma membrane and compacted (34). While there is no reliable way of judging parasite age in these fixed samples beyond the fact that they are 3 hpi or less, the presence of membranous foci could suggest that these parasites are either still in the process of disassembling their IMC and are therefore no more than 1 hpi (35) or developmentally delayed compared to WT parasites (Fig. 6C).

Overall, these data show that IMC1g-deficient (−ATc) parasites carry out successful and complete internalization as indicated by the formation of the parasitophorous vacuole, absence of residual tethering to the RBC membrane, and RESA staining of the RBC membrane. These parasites, however, do not show amoeboid form, the hallmark sign of normal morphology in the ring stage, or sustained remodeling of the RBC through the formation of a TVN. It is unclear to what extent parasites are metabolically active during initial RESA discharge and IMC disassembly. Absence of amoeboid form and sustained RBC remodeling are, however, clear signs that normal ring-stage physiologic processes are severely disrupted. Thus, we conclude that IMC1g-deficient parasites die (or are severely damaged and dying) within 1 h of internalization, and that the disappearance of infected RBCs from culture is downstream of parasite death.

### *Pf*IMC1c is a potential interacting partner of *Pf*IMC1g

While *Pf*IMC1g is the first alveolin confirmed to be essential in the blood stages of *P. falciparum*, it is only one of four alveolins which are upregulated during schizogony. Like *Pf*IMC1g, *Pf*IMC1c and 1e are also predicted to be essential in blood-stage asexual stages, while *Pf*IMC1f is predicted to be dispensable (16). To test these predictions, we attempted to directly knock out *Pf*IMC1e, 1c, 1g, and 1f by inserting constitutively expressed human dihydrofolate reductase (hDHFR), which conveys resistance to the drug WR99210, into their protein coding sequences, disrupting protein expression. Multiple attempts at knocking out *Pf*IMC1c, 1e, and 1g failed. However, we successfully knocked out *Pf*IMC1f without adverse effects for the parasites (Fig. S6B), confirming it is dispensable. To further investigate *Pf*IMC1c and 1e essentiality, we endogenously tagged them and added a TetR-DOZI knockdown system to control protein expression using ATc. While *Pf*IMC1c deficiency was lethal (Fig. S6A), induction of *Pf*IMC1e knockdown only had a small effect on parasite fitness likely due to insufficient KD (data not shown).

**Fig 7:**
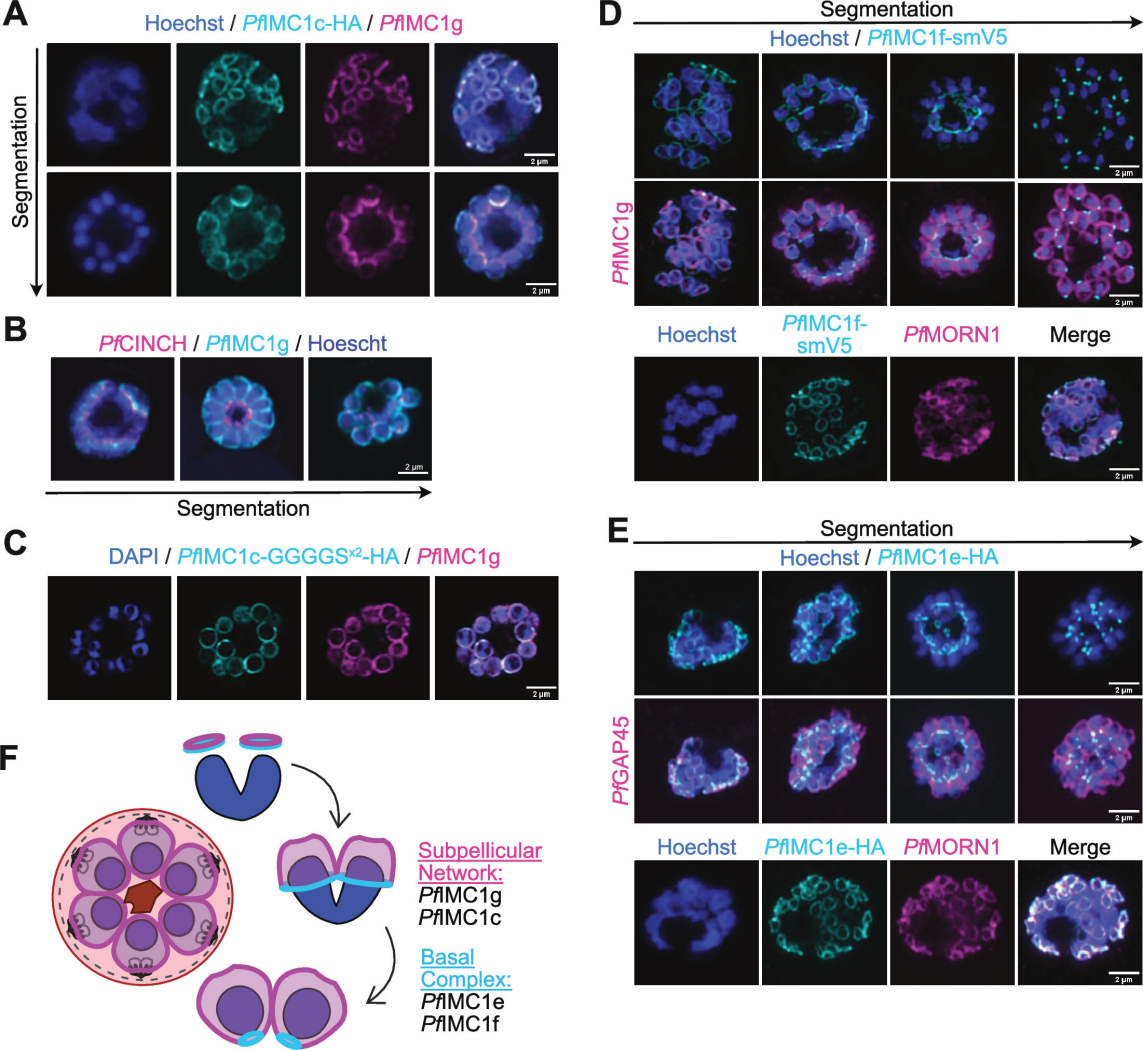
*Pf*IMC1c is a potential interacting partner of *Pf*IMC1g while *Pf*IMC1e and *Pf*IMC1f are novel members of the basal complex. (**A**) Batch IFA showing *Pf*IMC1c-HA (no linker) and *Pf*IMC1g. (**B**) Batch IFA showing *Pf*CINCH-smV5 as a basal complex marker and *Pf*IMC1g. (**C**) Batch IFA showing *Pf*IMC1c-HA (with linker) and *Pf*IMC1g. (**D**) Slide-based IFA showing *Pf*IMC1f-smV5 and IMC marker *Pf*IMC1g or basal complex marker *Pf*MORN1. (**E**) Slide-based IFA showing *Pf*IMC1e-HA and IMC marker *Pf*GAP45 or basal complex marker *Pf*MORN1. (**F**) Schematic of IMC and basal complex formation during schizogony and alveolin localization to each compartment. Scale bars = 2 µm.

Our tagged *Pf*IMC1c colocalized with *Pf*IMC1g, displaying a localization and distribution in the IMC identical to that previously reported (Fig. 7A). However, the presence of a c-terminal hemagglutinin (HA) tag on *Pf*IMC1c disrupts *Pf*IMC1g distribution. While parasites with untagged *Pf*IMC1c show an even distribution of *Pf*IMC1g throughout the IMC (Fig. 7B), c-terminally tagged *Pf*IMC1c parasites show basally enriched *Pf*IMC1g localization (Fig. 7A). This effect is variable from parasite to parasite, with the bulk of parasites showing normal *Pf*IMC1c and disrupted *Pf*IMC1g but with some parasites occasionally showing normal localization of both proteins or basal enrichment of both proteins. This effect is not noticeable at the beginning of segmentation but becomes more pronounced as segmentation progresses (Fig. 7A).

This matches our earlier observation that addition of a large tag to the *Pf*IMC1g c-terminus is deleterious for parasite health (Fig. S2) and suggests that the c-terminus of alveolins may in general be important for protein function or filament formation. Notably, adding a flexible linker between *Pf*IMC1c and its tag restored *Pf*IMC1g to its wild-type even distribution (Fig. 7C). This effect, coupled with their colocalization, suggests that *Pf*IMC1c and 1g could be interacting partners. To test this possibility, we also performed immunoprecipitation mass spectrometry analysis using *Pf*IMC1g as bait, which revealed *Pf*IMC1c as one of the most enriched proteins co-precipitated by *Pf*IMC1g, providing further evidence for their interaction (Fig. S7).

Intriguingly, *Pf*IMC1e and 1f were much less enriched, indicating that they are less closely associated with 1g. While *Pf*IMC1c had been localized to the IMC before, the localization of *Pf*IMC1e and 1f in schizonts remains unexplored. Thus, we sought to investigate the localization of epitope-tagged *Pf*IMC1e and 1f by super-resolution microscopy. To our surprise, they did not colocalize with *Pf*IMC1g (Fig. 7D and E). Rather, they remained at the leading edge of the IMC, expanding and contracting during schizogony in the same pattern as the basal complex. Furthermore, they both colocalize with the basal complex marker *Pf*MORN1 (36, 37). While there are several alveolins that localize to the basal complex of *T. gondii* (38), this is the first report of an alveolin that localizes to the basal complex of *Plasmodium*.

## DISCUSSION

In this work, we used iKD and iKO systems to confirm that the alveolin *Pf*IMC1g (PF3D7_0525800) is essential for *P. falciparum* asexual replication in RBCs. We went on to characterize its role in schizogony and invasion, the first functional characterization of an alveolin in the blood stages of *Plasmodium*. This characterization yielded multiple unexpected results. First, *Pf*IMC1g-deficient parasites only exhibit minor defects during segmentation, with most merozoites being indistinguishable from wild type by super-resolution fluorescence microscopy, ultrastructure expansion microscopy, and electron microscopy. So, unlike essential *P. berghei* alveolins studied in mosquito stages, *Pf*IMC1g only plays a minor role in determining merozoite cell shape. Second, despite being an essential member of the subpellicular network, *Pf*IMC1g does not play a key role in the formation and positioning of the subpellicular microtubules. Lastly, our live cell microscopy and expansion microscopy show that *Pf*IMC1g-deficient merozoites can complete all the steps of invasion up to RESA discharge and parasitophorous vacuole formation but cannot develop into amoeboid rings.

While these findings do not pinpoint a molecular cause of death, they are consistent with a model where IMC1g provides structural support during merozoite internalization. In mammalian cells which have suffered mechanical damage due to intermediate filament deficiencies, such damage often results in changes in membrane permeability and a loss of homeostasis inside the cell due to DNA damage and ion leakage that eventually leads to death (39). A loss in homeostasis of this kind is consistent with our observation that IMC1g-deficient parasites do not show expected signs of ring-stage metabolic activity. Thus, we suspect the key to *Pf*IMC1g’s molecular function lies in its effect on parasite biophysical properties.

Together with existing knowledge about the localization of *Pf*IMC1g, its homology to intermediate filaments, and alveolins’ role in tensile strength of *P. berghei* ookinetes, these results lead us to hypothesize that *Pf*IMC1g is a tension-bearing filament that provides elasticity to the IMC and protects merozoites from excessive deformation during invasion. This explains why slide-based IFAs on *Pf*IMC1g-deficient parasites, which involve smearing and drying parasites on a slide, result in parasites that appear distorted when compared to their wild-type control (Fig. S5B). Evidence of this increased deformability is also observed in our U-ExM and TEM images. Unfortunately, there is still a lack of tools to directly measure tensile strength and cell stiffness of *P. falciparum* merozoites due to their short lifespan outside the RBC. Specifically, *P. falciparum* merozoite infectivity has a half-life of 15 min at room temperature and centrifugation of merozoites leads to a large amount of agglutination and loss of infectivity (40). Developing a protocol to make biologically relevant measurements of merozoite tensile strength and cell stiffness therefore remains a difficult task.

**Fig 8:**
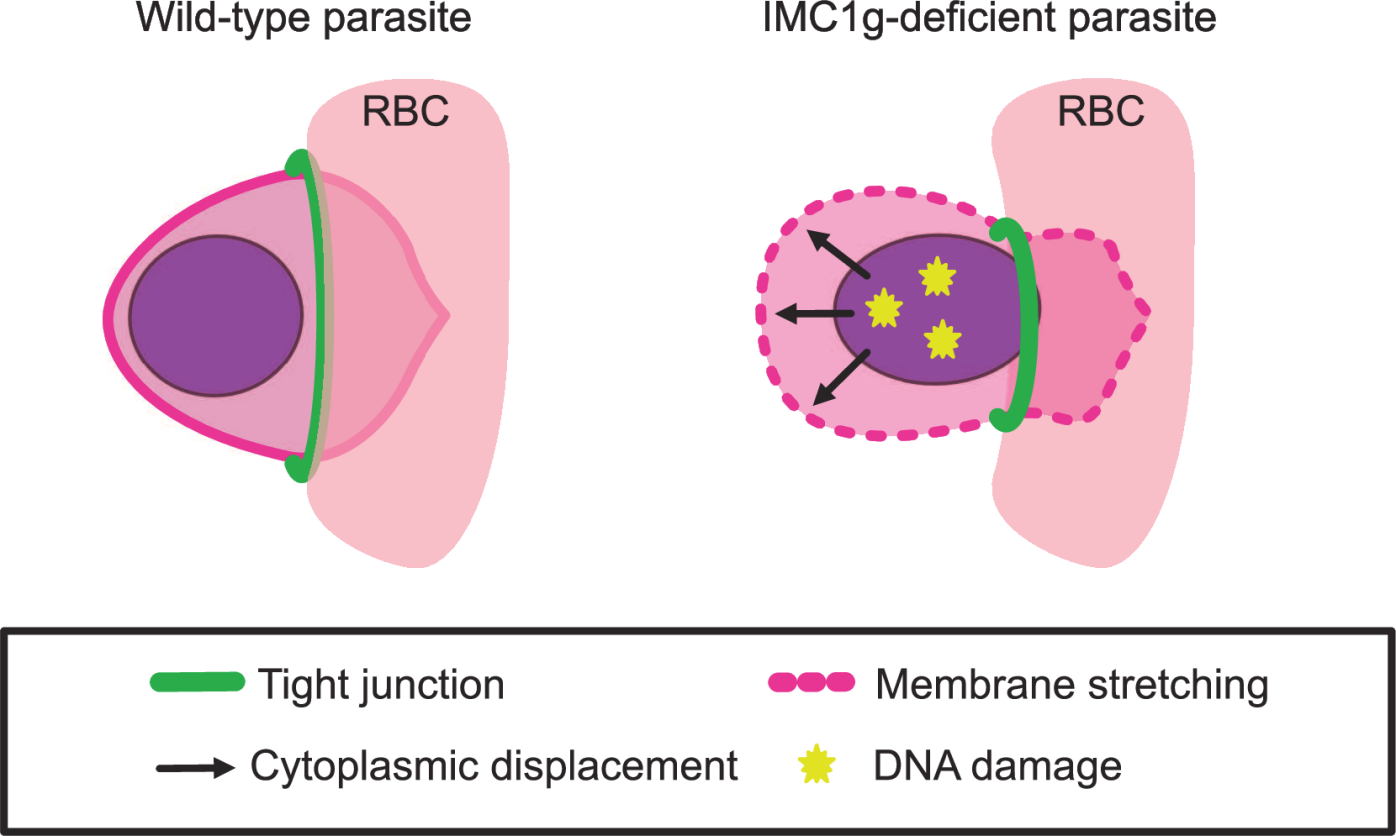
Diagram of hypothesized mechanism of death. Wild-type parasites have an elastic IMC that prevents parasite deformation by resisting cytoplasmic displacement. During invasion, the tight junction formed by the parasite enlarges as the parasite passes through. In *Pf*IMC1g-deficient parasites, the IMC membrane is less elastic. This leads to less resistance to cytoplasmic displacement. Thus, when parasites pass through the tight junction, they undergo a larger degree of deformation. This deformation can cause tears in the parasite membranes as they stretch to accommodate displaced cytoplasm and DNA damage due to shear stress.

A loss in IMC elasticity can also explain why we observe a more severe cell shape phenotype in ookinetes and sporozoites than in merozoites. In ookinetes and sporozoites, the nucleus adopts a flattened shape to conform to the restrictions of a narrow cell body. This is likely facilitated by the elastic, rope-like properties of alveolins which return the IMC to its narrow shape and prevent deformation. In the absence of essential ookinete and sporozoite alveolins, the IMC loses its elasticity, causing the area of the IMC surrounding the nucleus to bulge (5, 12, 13). Our model for damage during invasion follows similar principles; if the IMC is more deformable, parasites would have less resistance against cytoplasmic displacement, resulting in a smaller tight junction and increased shear stress on organelles (Fig. 8).

In addition to our functional characterization of *Pf*IMC1g, we report that the alveolin composition of the merozoite SPN is much less diverse than that of the ookinete and sporozoite SPN: while four alveolins are expressed at the schizont and merozoite stage, only two, *Pf*IMC1c and 1g, localize to the SPN. The other two alveolins, *Pf*IMC1e and 1f, localize to the basal complex. Alveolins are a known component of the *Toxoplasma* basal complex and *Pb*IMC1e was previously observed to enrich basally in ookinetes, but this is the first report of an alveolin localizing specifically and exclusively to the basal complex in *Plasmodium*.

The above observations have opened a new framework for understanding the role of alveolins in SPN biogenesis, function, and architecture. For example, studies performed in the mosquito stages of *Plasmodium* have observed that absence of certain alveolins leads to a variety of defects and that double knockout of alveolins exacerbates some phenotypes but not others (13). These studies have so far been unable to conclusively determine to what extent these phenotypes were interdependent or a result of alveolin redundancy. In this work, we were able to observe that loss of merozoite infectivity as a result of *Pf*IMC1g depletion is independent of cell morphological abnormalities, demonstrating that cell morphology does not necessarily lie upstream of other alveolin deficiency phenotypes. We also confirm that both alveolins in the merozoite SPN, *Pf*IMC1g and 1c, are essential and that they are potential interacting partners. Thus, it is a possibility that simultaneous disruption of *Pf*IMC1g and 1c could result in more dramatic segmentation defects in merozoites. Our ongoing research attempts to determine whether *Pf*IMC1c and 1g form distinct filaments, shedding light on whether *Pf*IMC1g has partial redundancy with *Pf*IMC1c.

While our system could have presented an opportunity to assess whether the motility defects caused by alveolin knockouts are independent from cell shape defects, our attempts to assess merozoite gliding motility have thus far been unable to obtain reproducible results. Therefore, we were unable to measure a motility defect, or lack thereof, in our *Pf*IMC1g-deficient parasites.

Overall, we show that *Pf*IMC1g is essential and demonstrate its importance for RBC invasion. While we could not conclusively pinpoint the molecular mechanism behind parasite death, our data bring valuable insights into the stage-specific roles of alveolins. Our phenotypic observations and mapping of *Pf*IMC1e and 1f to the basal complex open new avenues of research to better understand the interactions and specialized functions of these filaments in the asexual blood stages.

## MATERIALS AND METHODS

### Accession numbers

Accession numbers are as follows: *Pf*IMC1g, PF3D7_0525800; *Pf*IMC1c, PF3D7_1003600; *Pf*IMC1f, PF3D7_1351700; *Pf*IMC1e, PF3D7_0304100.

### *Plasmodium falciparum* culture

The 3D7 strain of *P. falciparum*, obtained from the Walter and Eliza Hall Institute (Melbourne, Australia), and the 3D7(pfs47)DiCre strain, obtained from from Ellen Knuepfer at The Francis Crick Institute, were cultured in RPMI-1640 (Sigma) supplemented with 25 mM HEPES [4-(2-hydroxyethyl)-1-piperazineethanesulfonic acid] (EMD Biosciences), 0.21% sodium bicarbonate (Sigma), 50 mg/L hypoxanthine (Sigma), 0.5% Albumax (Invitrogen), and 2 mM choline chloride (Sigma). Parasites were maintained in human O+ erythrocytes at 4% hematocrit. Packed erythrocytes were obtained from Valley Biomedical. Unless otherwise indicated, all parasite cultures (including transfection, parasite maintenance, and all experimental assays) were done under shaking conditions.

### Parasite synchronization

Parasites were synchronized using one or a combination of the following approaches. Schizont-stage parasites were isolated by density centrifugation with 60% (vol/vol) Percoll (GE Healthcare), washed in complete media, and allowed to re-invade fresh erythrocytes. Rings were selected by incubation in 5% (wt/vol) sorbitol (Sigma-Aldrich) for 10 min followed by a wash in complete media. Invasion was constrained to a desired window by blocking merozoite attachment with 125 µg/mL heparin (Sigma) when invasion was not desired.

### Parasite transfection and new line generation

#### IMC1g^smV5^ iKD

One hundred micrograms of homology-directed repair (HDR) plasmid was linearized by digestion, puriﬁed, and co-transfected with 100 µg Cas9 targeting plasmid into 3D7 parasites. Parasites were maintained on 500 nM ATc and drug pressure was applied 1 day post-transfection with 2.5 nM WR99210 (Jacobus Pharmaceuticals).

#### IMC1g iKD

One hundred micrograms of HDR plasmid was linearized by digestion, puriﬁed, and co-transfected with 100 µg Cas9 targeting plasmid into 3D7(*pfs*47)DiCre parasites (18). Parasites were maintained on 500 nM ATc and drug pressure was applied 1 day post-transfection with 2.5 nM WR99210.

#### IMC1g^HAx2^ iKO

One hundred micrograms of HDR plasmid was linearized by digestion, puriﬁed, and co-transfected with 100 µg Cas9 targeting plasmid into 3D7(*pfs*47)DiCre parasites (18). Parasites were maintained on 500 nM ATc and drug pressure was applied 1 day post-transfection with 2.5 nM WR99210.

#### IMC1c^HAx2^ iKD

One hundred micrograms of HDR plasmid was linearized by digestion, puriﬁed, and co-transfected with 100 µg Cas9 targeting plasmid into 3D7(*pfs*47)DiCre parasites (18). Drug pressure was applied 1 day post-transfection with 2.5 nM WR99210.

#### IMC1c^Linker-HAx2^ iKD

One hundred micrograms of HDR plasmid was linearized by digestion, puriﬁed, and co-transfected with 100 µg Cas9 targeting plasmid into 3D7(*pfs*47)DiCre parasites (18). Parasites were maintained on 500 nM ATc and drug pressure was applied 1 day post-transfection with 2.5 µg/mL blasticidin (Research Products International, RPI).

#### IMC1e^HAx2^ iKD

One hundred micrograms of HDR plasmid was linearized by digestion, puriﬁed, and co-transfected with 100 µg Cas9 targeting plasmid into 3D7(*pfs*47)DiCre parasites (18). Parasites were maintained on 500 nM ATc and drug pressure was applied 1 day post-transfection with 2.5 nM WR99210.

#### IMC1f^smV5^

One hundred micrograms of HDR plasmid was linearized by digestion, puriﬁed, and co-transfected with 100 µg Cas9 targeting plasmid into 3D7(*pfs*47)DiCre parasites (18). Parasites were maintained on 500 nM ATc and drug pressure was applied 1 day post-transfection with 2.5 nM WR99210.

#### IMC1f KO

One hundred micrograms of HDR plasmid was linearized by digestion, puriﬁed, and co-transfected with 100 µg Cas9 targeting plasmid into 3D7(*pfs*47)DiCre parasites (18). Drug pressure was applied 1 day post-transfection with 2.5 nM WR99210.

#### CINCH^smV5^ iKD

Strain was generated as previously described (4).

Integration of each targeting construct was conﬁrmed by PCR. Tet aptamer size was conﬁrmed by amplifying the aptamer region and digesting the PCR fragment with PspOMI and KpnI. Individual transgenic clones for PfIMC1g^smV5^ iKD, PfIMC1g iKD, PfIMC1g^HAx2^ iKO, PfIMC1c^HAx2^ iKD, PfIMC1e^HAx2^ iKD, and PfIMC1f KO parasite lines were obtained by limiting dilution. Integration PCR descriptions are provided in Table S1 and Fig. S8.

### Washout of ATc for protein knockdown

Depletion of PfIMC1g was achieved by adjusting a tightly synchronized ring culture to the desired parasitemia and then washing it three times in ATc-free RPMI. The washed culture was then divided into two dishes with half of the synchronized parasites receiving 500 nM ATc, and the other half receiving 0 nM ATc.

### Depletion of PfIMC1g by inducible knockout

A tightly synchronized ring culture was adjusted to the desired parasitemia and then divided into two dishes with half of the synchronized parasites receiving 100  nM rapamycin (VWR), and the other half receiving dimethyl sulfoxide (DMSO) as a vehicle control. The final concentration of DMSO in both conditions was 0.01%.

### Reagents and antibodies

Primers were obtained from Integrated DNA Technologies or Life Technologies; restriction enzymes were obtained from New England Biolabs (NEB).

#### Small molecules

The name and concentration of small molecules used in this study are as follows: compound 1 (C1) {4-[2-(4ﬂuorophenyl)-5-(1-methylpiperidine-4-yl)−1H-pyrrol-3-yl]pyridine} (50 mM stock in DMSO, 2.5 µM working concentration), E64 (ThermoFisher, 10 mM stock in DMSO, 10 µM working concentration), WR99210 (Jacobus Pharmaceuticals; 2.5 nM working concentration), and anydrotetracycline (Cayman Chemical, 0.5 µM working concentration).

#### Primary antibodies

Commercially available antibodies were obtained from Sigma (rat anti-HA [clone 3F10]), DSHB (mouse anti-tubulin [12G10]), Bio-Rad (mouse anti-V5), and Immunology Consultant Laboratories (rabbit anti-V5, RV5-45A-Z). Other antibodies were kindly provided by Robin Anders at The Walter & Eliza Hall Institute of Medical Research (mouse anti-PfAMA1 clone 1F9, mouse anti-RESA clone 28/2) (41), Julian Rayner at Cambridge Institute for Medical Research (rabbit anti-PfGAP45) (42), Anthony Holder at MRC National Institute for Medical Research (mouse anti-PfMSP1, clone 1E1) (24), Matt Dixon at the University of Melbourne (rabbit anti-PhIL1) (23), and Michael Makler at Flow Inc. (mouse anti-PfLDH).

#### Secondary antibodies

Secondary antibodies from ThermoFisher were as follows: Goat anti-Rat IgG (H+L) Cross-Adsorbed Secondary Antibody, Alexa Fluor 488 (A11006), Goat anti-Rabbit IgG (H+L) Highly Cross-Adsorbed Secondary Antibody, Alexa Fluor 488 (A11034), Goat anti-Mouse IgG (H+L) Highly Cross-Adsorbed Secondary Antibody, Alexa Fluor 488 (A11029), Goat anti-Rabbit IgG (H+L) Highly Cross-Adsorbed Secondary Antibody, Alexa Fluor 555 (A21429), Goat anti-Mouse IgG (H+L) Highly Cross-Adsorbed Secondary Antibody, Alexa Fluor 594 (A11032), Goat anti-Rabbit IgG (H+L) Highly Cross-Adsorbed Secondary Antibody, Alexa Fluor 594 (A11037). Secondary antibodies from LI-COR were as follows: IRDye 680RD Donkey anti-Rabbit IgG Secondary Antibody, IRDye 680RD Donkey anti-Mouse IgG Secondary Antibody, IRDye 800CW Goat anti-Mouse IgG Secondary Antibody, IRDye 800CW Goat anti-Rabbit IgG Secondary Antibody.

#### Other stains

Other stains used were as follows: Hoechst (Invitrogen, Catalog no. H3570; 1 µg/mL), Alexa Fluor 568 NHS ester in DMSO (Lumiprobe Cat. No. 14820; 10 µg/mL), SYTOX Deep Red (ThermoFisher Cat. No. S11381; 1 µM).

### Plasmid construction

All PCRs were performed with PrimeSTAR GXL DNA Polymerase (Takara Bio). All sequences for oligonucleotides and gene block sequences are provided in Table S1. Cloning outline for each plasmid is described in Table S1. All transformations were performed using *Escherichia coli* XL-10 Gold chemically competent bacteria.

### Genomic DNA extraction

Parasite genomic DNA was isolated from 5 mL of ~2% parasitemia schizont-stage culture using the EZ-10 Spin Column Blood Genomic DNA Miniprep Kit (Bio Basic).

### Growth assays

After ring-stage washout or addition of rapamycin, synchronized parasite lines and their parental lines were diluted to 0.25% parasitemia at 1% hematocrit. One hundred microliters of culture was collected 2 and 4 days after plating and immediately stained in 1:1,000 dilution of SYBR green I (Invitrogen) in 0.5% bovine serum albumin phosphate-buffered saline (BSA-PBS) for 20 min at room temperature. Stained parasites were washed in 0.5% BSA-PBS and resuspended in filtered PBS. The proportion of infected cells was measured by ﬂow cytometry. One hundred thousand RBCs were counted for each condition. Data represented as mean values ± standard deviation (SD) of three independent biological replicates each with three technical replicates.

### Generation of primary antisera

Polyclonal rabbit antisera against amino acids 176–189 of PF3D7_0525800/IMC1g (KEEIPINELKENQT) conjugated to keyhole limpet hemocyanin were generated and affinity purified (Genscript).

### Immunoblots

Parasite pellets were purified by lysing RBCs using 0.05% saponin in PBS with protease inhibitors (SigmaFast Protease Inhibitor Cocktail, Sigma). Pellets were then resuspended in Laemmli buffer and incubated for 10 min at 95°C. Equal volumes of each paired sample was run on a 4%–20% Tris-glycine-sodium dodecyl sulfate gel and transferred to a nitrocellulose membrane. Membranes were blocked in Odyssey PBS blocking buffer (LI-COR Biosciences), incubated overnight at 4°C with primary antibody diluted in blocking buffer, and then incubated with secondary antibodies diluted in blocking buffer. Membranes were scanned on a Licor Odyssey CLx imager system. Data shown are representative of three independent biological replicates.

Dilutions for primary antibodies were as follows: 1:1,000 for anti-IMC1g or 1:2,000 for anti-PfLDH. All secondary antibodies were used at 1:2,000.

### Immunoprecipitation and mass spectrometry

Synchronized parasites were expanded to 200 mL at 2% schizontemia per condition and collected at approximately 48 hpi. At collection, RBCs were lysed with 0.05% saponin in PBS with protease inhibitors (SigmaFast Protease Inhibitor Cocktail, Sigma). Parasite pellets were lysed in RIPA (50 mM Tris-HCl, pH 7.5, 150 mM NaCl, 1% NP-40, 0.5% sodium deoxycholate, 0.1% sodium dodecyl sulfate) plus protease inhibitor for 30 min on ice, sonicated twice at 20% amplitude for 30 s, and insoluble material was removed by centrifugation. Parasite lysate was applied to magnetic α-V5 beads (MBL International) and incubated for 3 h at 4°C. Beads were washed three times with fresh RIPA plus protease inhibitors, resuspended in 50 µL of 5 mM ammonium bicarbonate, and submitted for detergent removal, on-bead digestion, and mass spectrometry analysis. Mass spectrometry results were analyzed by comparing the number of unique and total peptides present in experimental and control samples. Data shown represent peptides detected in two independent biological replicates.

### Nanoluciferase assay

Synchronized (2% schizonts) parasite culture was resuspended and 300 µL was transferred to a 1.5 mL Eppendorf. Media was removed by centrifugation and discarded. Fumarizine (final concentration of 44 µM) was mixed with nLuc buffer (100 mM MES [pH 6], VWR 102587-278; 1 mM CDTA-H20, Sigma 319945-25G; 0.5% [vol/vol] Tergitol NP40s, Sigma NP40S-100ML; 0.05% [wt/vol] Pluronic F-127, Sigma P2443-250G; 150 mM KCL, RPI P41000-1000.0; 1 mM DTT, Sigma D0632-5G; 35 mM Thiourea, Sigma T8656-50G) and 300 µL was added to the parasite pellet. The resulting lysate was transferred to a white bottom 96-well plate in 100 µL triplicates. Luminescence was read using a SpectraMax iD3 Microplate reader. Data represented as mean values ± SD of three independent biological replicates.

### IFAs

Tightly synchronized parasite lines (after ring-stage washout when appropriate) were seeded at 2%–10% ring parasitemia and 1%–4% hematocrit depending on the assay and parasite mass required. Parasites were given daily media changes and, for experiments observing AMA1 translocation, arrested using E64 2–3 h prior to harvest.

#### Slide-based IFAs

Dried blood smears were fixed with 4% PFA for 10 min. Following ﬁxation, slides were washed three times in PBS and permeabilized with 0.1% Triton X-100 in PBS for 10 min. Parasites were then washed three times in PBS and blocked in 3% BSA-PBS for 1 h at room temperature or overnight at 4°C. Primary antibodies were diluted in 3% BSA-PBS, and slides were incubated overnight at 4°C. Slides were washed three times in PBS, and secondary antibodies were diluted in 3% BSA-PBS and applied for 45 min at room temperature. Slides were washed three times in PBS and mounted with VECTASHIELD Vibrance with DAPI (4´,6-diamidino-2-phenylindole) or washed once and stained with Hoechst (1 µg/mL) in PBS for 10 min at room temperature (RT). Slides stained with Hoechst were washed three times in PBS and mounted with VECTASHIELD Vibrance or PLUS (Vector Laboratories).

#### Batch IFAs

One hundred microliters of infected RBCs was transferred to an Eppendorf tube, spun down to remove media, and washed once in 1 mL PBS. Parasites were then fixed in 4% PFA/0.0075% glutaraldehyde (PFA/GA) in PBS for 30 min at RT. Parasites were washed once in PBS and then permeabilized with 0.1% Triton X-100 in PBS for 10 min. Parasites were washed once in PBS, and then free aldehydes were quenched with 0.1 mg/mL sodium borohydride in PBS for 10 min to decrease background fluorescence. Parasites were washed twice in PBS and then blocked in 3% BSA-PBS for 1 h at room temperature or overnight at 4°C. Parasites were spun down and resuspended in primary antibody solution (diluted in 3% BSA-PBS) and incubated overnight at 4°C. After washing three times in 3% BSA-PBS, parasites were resuspended in secondary antibody solution (diluted in 3% BSA-PBS) and incubated for 45 min at RT protected from light. Hoechst 4× solution in PBS was added to the parasites in secondary antibody solution to obtain a final concentration of 1 µg/mL Hoechst and then incubated for an additional 10 min. Parasites were washed two times in 3% BSA-PBS and then resuspended in 100 µL of PBS. Resuspended parasites were settled on poly-D-lysine-coated (Gibco) coverslips for 15–20 min at RT protected from light and then washed two times by immersion in PBS. Parasites were then mounted in VECTASHIELD Vibrance or PLUS media and sealed with nail polish. All centrifugations were carried out for 2.5 min at 300 × *g*.

Cells were visualized on a Zeiss LSM880 with Airyscan or Zeiss LSM980 with Airyscan2 (Plan Apo 63×/1.4 Oil DIC III) for super-resolution microscopy. Dilutions for primary antibodies were as follows: mouse anti-PfRON4 1:200; mouse anti-V5 1:200; rabbit anti-PfGAP45 1:5,000; rabbit anti-PfMORN1 1:1,000; mouse anti-PfMSP1 1:500; mouse anti-PfAMA1 1:200; rat anti-HA 3F10 1:100; rabbit anti-IMC1g 1:2,500.

### Ultrastructure expansion microscopy

Parasites were magnetically (MACS) purified (for expansion on schizonts) and allowed to recover in complete media for at least 1 h at 37°C. For “post-egress” schizonts, WT and *Pf*IMC1g-deficient parasites were arrested for 2–3 h with E64 prior to harvesting. Alternatively, for expansion on invaded RBCs, a culture of highly synchronized parasites was prepared as described for the post-invasion Field’s stain time course and adjusted to 1% hematocrit (HCT) 3 h post-invasion using complete RPMI. Parasites were then sedimented on poly-D-lysine-coated (Gibco) coverslips for 30 min at 37°C and fixed in warmed 4% PFA for 20 min and prepared for U-ExM as previously described (43, 44). Briefly, coverslips were washed in warmed PBS after fixation and incubated in formaldehyde (FA)/acrylamide (AA) mix (1.4% FA and 2% AA) overnight at 37°C. This was followed by gelation in 0.5% ammonium persulfate (APS)/0.5% tetramethylethylenediamine (TEMED)/monomer solution (19% sodium acrylate; 10% AA; 0,1% *N,N’*-methylenbisacrylamide (BIS-AA) in PBS 10×) for 1 h at 37°C. Gel-embedded parasites were then incubated for 90 min in denaturation buffer (200 mM SDS, 200 mM NaCl, 50 mM Tris, pH 9) at 90°C and expanded in MiliQ water. Gels were shrunk in PBS and then blocked in 3% BSA-PBS for 30 min or overnight at 4°C. Parasites were stained with primary antibody against alpha tubulin (1:500) or against RESA (1:100) diluted in 3% BSA-PBS overnight at 4°C. Gels were washed 3 × 10 min in PBS-0.1% Tween prior to incubation in secondary antibody solution (PBS, anti-mouse Alexa 488 at 1:500, SYTOX Deep Red at 1:1,000, and either 568 NHS-ester at 10 µg/mL or 405 NHS-ester at 10 µg/mL and BODIPY-TRc at 2 µM) for 2.5 h at RT. This was followed by three washes of 10 min in PBS-Tween and a second round of expansion in water before imaging. Gels were mounted on poly-D-lysine-coated 35 mm coverslip bottomed dishes (Cellvis NC0409658, Fisher Scientific) and visualized on a Zeiss LSM980 or Zeiss LSM900 with Airyscan2.

### Time-lapse microscopy

After ring-stage washout, tightly synchronized parasite lines were seeded at 10%–12% parasitemia and 1% hematocrit. Parasites were given daily media changes and arrested using C1 2 h prior to harvest. Five hundred microliters of each culture were collected and washed twice with warm media to release from C1. Parasites were settled on dishes immediately and imaged using brightfield every 4 s using a Nikon Ti Eclipse inverted microscope. For DIC imaging of egressed merozoites without RBCs, parasites underwent the same preparation but were MACS purified prior to C1 addition, recovered for 1 h in warm complete media, and settled on ibidi μ-slide I Luer chambers (Cat. No. 80176) after C1 release. Samples were imaged using a Nikon Ti2-E inverted microscope 1 h post C1 release.

For DIC imaging of invaded RBCs, tightly synchronized parasite lines were washed from ATc as rings and seeded at 8%–10% parasitemia and 2% hematocrit. To constrain invasion to a 3-h time window, 125 µg/mL heparin (Sigma) was used to prevent parasite invasion starting at 24 h pre-egress. Then, 2 h pre-egress, C1 was added to each parasite culture. After 2–3 h of C1 incubation, heparin and C1 were washed out and parasites were allowed to invade for 3 h under shaking conditions. Then, 250 µL of culture was collected and adjusted to 1% HCT using phenol red-free media supplemented with Trolox (Acros Organics, final concentration 10 µM) and 5 µg/mL Hoechst. After incubating for 10 min protected from light, 30 µL of sample was settled on a four-chamber 35 mm glass bottom dish (Cellvis) coated with concanavalin A (Sigma). After settling for 10 min at 37°C, 400 µL of phenol red-free media supplemented with Trolox (10 µM) was added to each chamber. Regions of interest (ROIs) were selected using Hoechst and brightfield. Each ROI contained at least one RBC meeting the following criteria: single nucleus as observed by Hoechst, no extracellular parasite material attached to RBC, and no hemozoin visible by brightfield. After acquiring an initial picture with both Hoechst and DIC, parasites were imaged every 30 s for 30 min using DIC only.

### Transmission electron microscopy

E64-treated WT and PfIMC1g-deficient schizonts were purified by MACS. The parasite pellet was washed with 1 mL incomplete RPMI, and resuspended in 50 µL incomplete RPMI. Then, 50 µL fixative (2.5% paraformaldehyde, 5% GA, 0.06% picric acid in 0.2 M cacodylate buffer) was added to the pellet. Fixed pellets were submitted to the Harvard Medical School Electron Microscopy Core. There, they were washed once in 0.1 M cacodylate buffer, twice in water, and then incubated for 1 h in 1% osmium tetroxide/1.5% potassium ferrocyanide in water. Next, the samples were washed twice in water then once in 50 mM maleate buffer pH 5.15 (MB). Samples were incubated for 1 h in 1% uranyl acetate in MB, then washed once in MB and twice in water. Pellets were then subjected to dehydration by increasing concentrations of ethanol and were put successively in 50%, 70%, 90%, 100%, and 100% ethanol for 10 min each. Following dehydration, samples were incubated in propylene oxide for 1 h, then overnight with a 1:1 mixture of propylene oxide and TAAB 812 Resin (TAAB, #T022). The next day, samples were embedded in TAAB 812 Resin then polymerized at 60C for 48 h. Slices were imaged using a JEOL 1200EX - 80kV electron microscope. For analysis, images were blinded and loaded onto ImageJ. Parasite roundness was quantified by thresholding parasites and then applying the shape descriptors measurement under the Analyze menu in ImageJ.

### Measuring daughter cell size

Images were blinded and then the largest diameter of each cell (estimated by eye) was traced using the polygon selection tool on FIJI and saved to the ROI manager. The area of a fitted ellipse was calculated by measuring the Ferret’s minimum and maximum diameter and plugging them into the formula for the area of an ellipse ( $A=\pi r_{min}r_{max}$ ). All datapoints collected and their mean values across three independent biological replicates are shown.

### Measuring tubulin and cell length

Images were blinded and then, showing the NHS-ester channel, the apical polar ring and basal complex of each parasite were mapped with the point tool and saved to the ROI manager on ImageJ. Then, showing the tubulin channel only, the beginning and end of each subpellicular microtubule bundle were mapped and saved to the ROI manager. The coordinates of each point were then exported and the distances between each pair of coordinates (APR and basal complex for cell length and tubulin for the subpellicular microtubules) were determined by calculating their hypotenuse in 3D space $(D=\sqrt{{\left(x_{1-}x_{2}\right)}^{2}+{\left(y_{1-}y_{2}\right)}^{2}+{\left(z_{1-}z_{2}\right)}^{2}})$ .

### Post-invasion Field’s stain time course

After ring-stage washout, tightly synchronized parasite lines were seeded at 2% parasitemia and 4% hematocrit. To constrain invasion to a 3-h time window, 125 µg/mL heparin (Sigma) was used to prevent parasite invasion starting at 24 h pre-egress. Then, 2 h pre-egress, C1 was added to each parasite culture. After 2 h of C1 incubation, heparin and C1 were washed out and parasites were allowed to invade for 3 h. Smears for Field’s stains were collected and stained with Hemacolor every hour. At the end of 3 h, heparin was added again to inhibit further invasion. Smears for Field’s stains were collected and stained once more at 20 hpi. Randomized ROIs were captured on an Olympus BX40 microscope. Images were blinded and then each parasite was categorized according to the morphologies described in Table S1. Tallies were kept using the cell counter tool on ImageJ. Data represented as mean values ± SD of three independent biological replicates.

## Data Availability

All data generated during this study are included in the manuscript and supplementary files. Protocols, raw data, or any materials used in this study are available upon request.

## SUPPLEMENTAL MATERIAL

Supplemental Figures mbio.01507-23-s0001.pdf Fig. S1 to S8.

Video Legend mbio.01507-23-s0002.docx Legend for Video S1.

Supplemental Tables mbio.01507-23-s0003.xlsx Tables S1 to S4.

Video S1 mbio.01507-23-s0004.avi Video microscopy of newly invaded PfIMC1g-sufficient (+ATc) and PfIMC1g-deficient (-ATc) parasites.

ASM does not own the copyrights to Supplemental Material that may be linked to, or accessed through, an article. The authors have granted ASM a non-exclusive, world-wide license to publish the Supplemental Material files. Please contact the corresponding author directly for reuse.
