# Supplementary material for: Essential function of alveolin PfIMC1g in the Plasmodium falciparum asexual blood stage
Source: mBio. 2023 Sep 15;14(5):e01507-23. doi: 10.1128/mbio.01507-23 (PMC10653860; doi:10.1128/mbio.01507-23)
Supplement: Supplemental Figures — Fig. S1 to S8. [file mbio.01507-23-s0001.pdf]

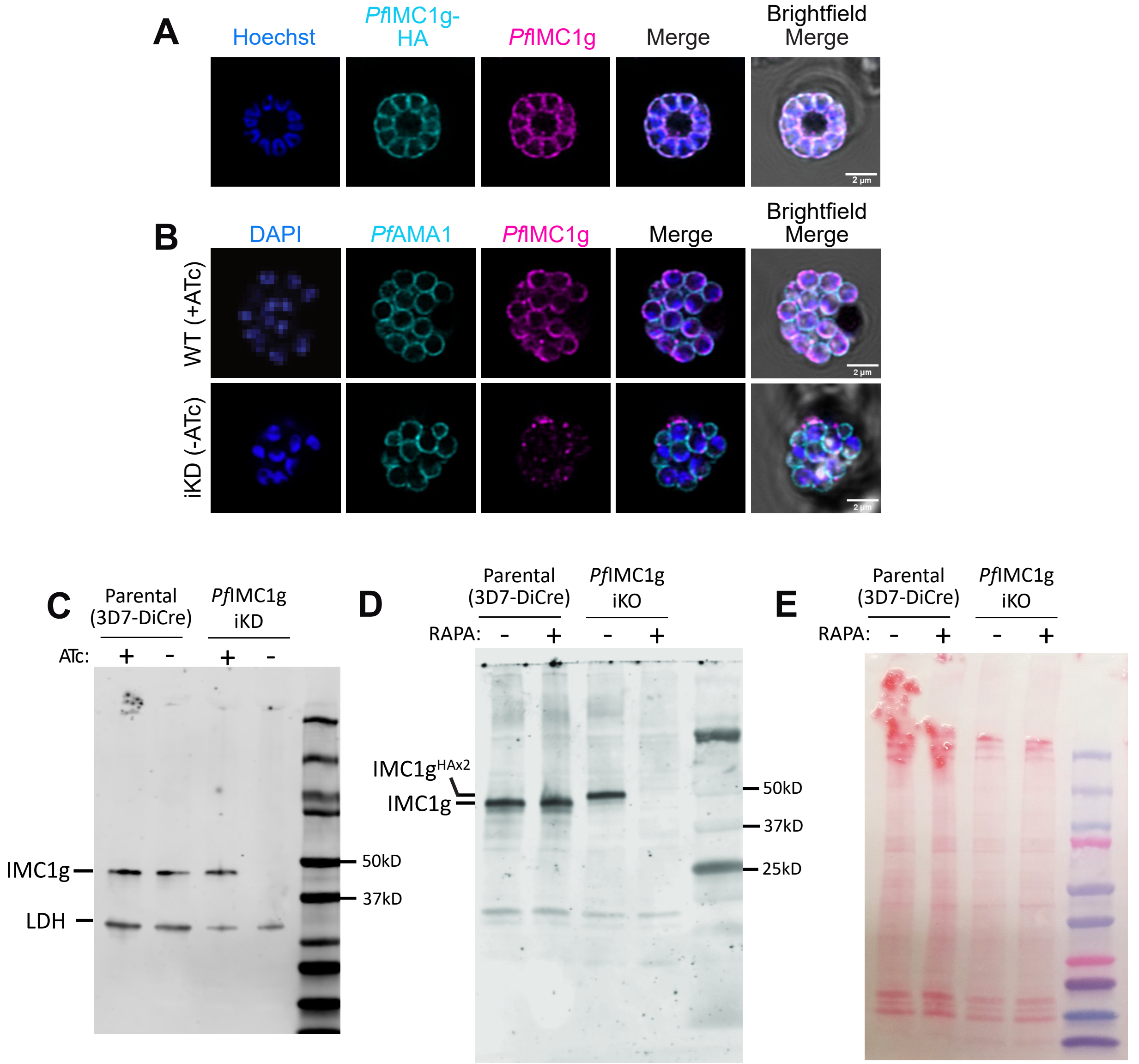

Supplemental Figure 1. Validation of *Pf*IMC1g primary antibody and iKD/iKO systems. A) Batch IFA of *Pf*IMC1g-HA parasites comparing staining of anti-HA vs anti-*Pf*IMC1g primary antibody. **B)** Batch IFA showing distribution of *Pf*IMC1g in WT (+ATc) vs KD (-ATc) parasites. **C)** Western blot showing the level of protein knockdown in *Pf*IMC1g iKD parasites vs. their parental line in the presence and absence of ATc. Top bands, *Pf*IMC1g primary. Bottom bands, *Pf*LDH loading control. **D)** Western blot showing the level of protein loss in *Pf*IMC1g iKO parasites vs their parental line in the presence and absence of RAPA. **E)** Loading control of gel in panel D using Ponceau as a general protein stain. Scale bars = 2µm.

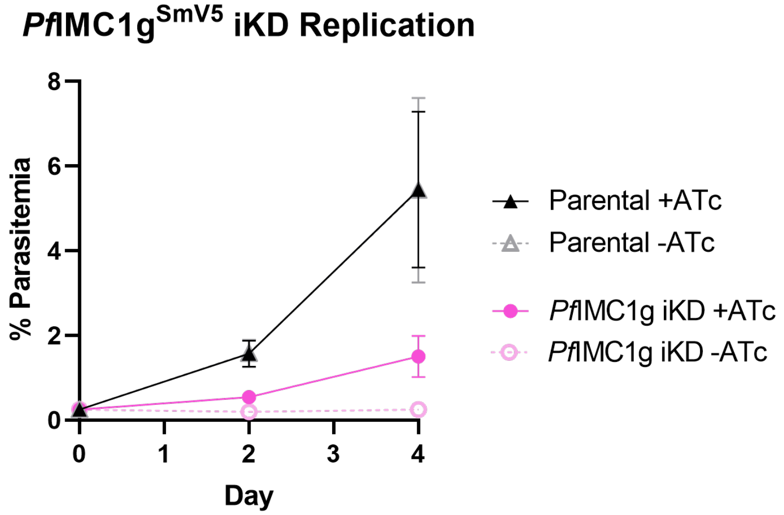

Supplemental Figure 2. Large C-terminal tag disrupts *Pf*IMC1g function. Replication of *Pf*IMC1g-smV5 WT (+ATc) and KD (-ATc) parasites, in pink circles, and their parental line, in black triangles, as measured by flow cytometry. Graph shows mean ± SD of 3 biological replicates.

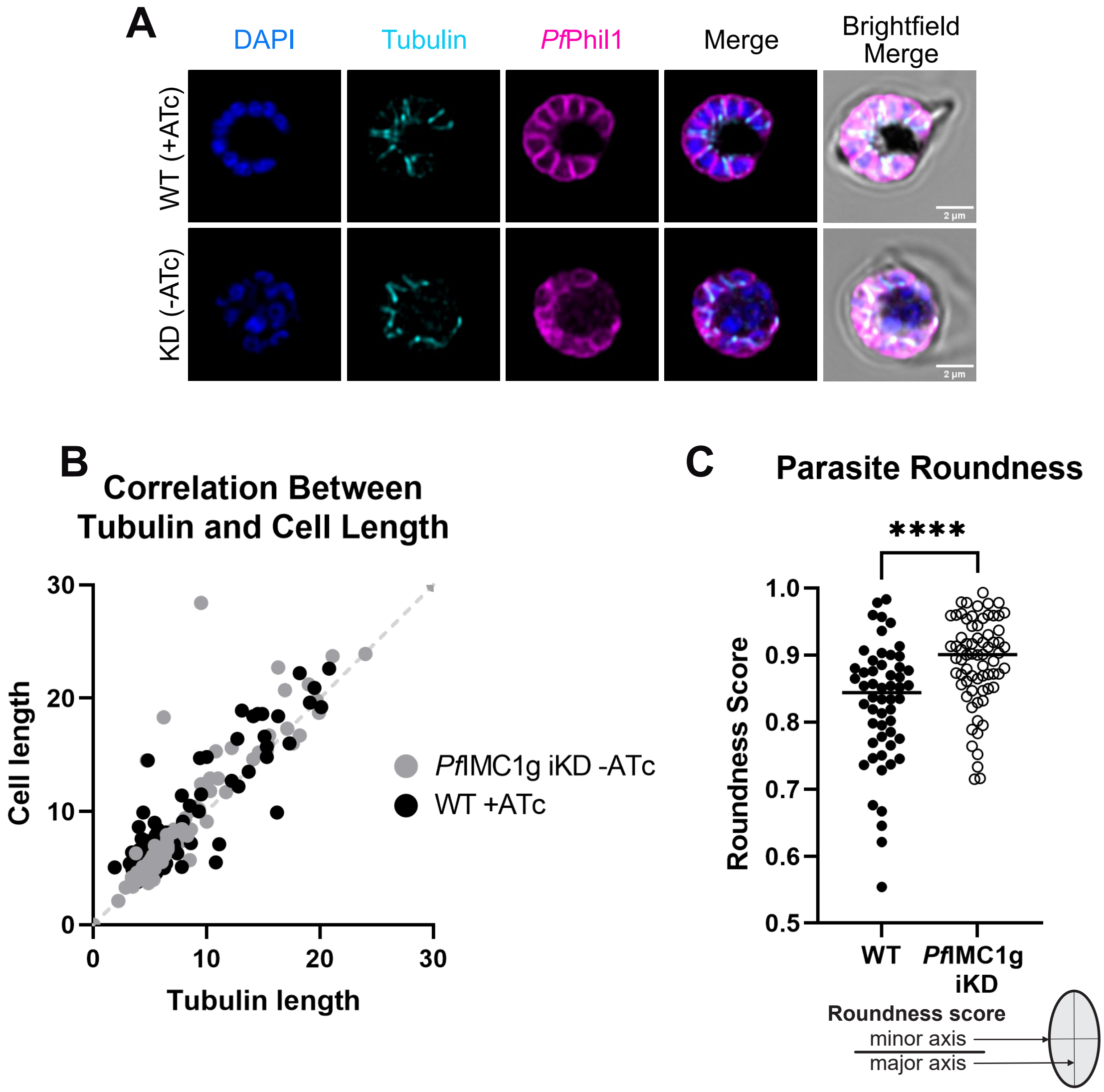

Supplemental Figure 3. *Pf*IMC1g-deficient parasites lack abnormalities in their subpellicular microtubules but appear rounder by TEM. A) Batch IFA showing tubulin and IMC marker *Pf*PhIl1 in schizonts under *Pf*IMC1g WT (+ATc) and KD (-ATc) conditions. Scale bar = 2µm. **B)** Correlation between subpellicular microtubule length and merozoite length (in µm), pooled data from expansion microscopy reconstructions of five schizonts. **C)** Quantification of merozoite roundness in TEM images. Each point represents the roundness score of an individual daughter cell; WT n=54, iKD n=70.

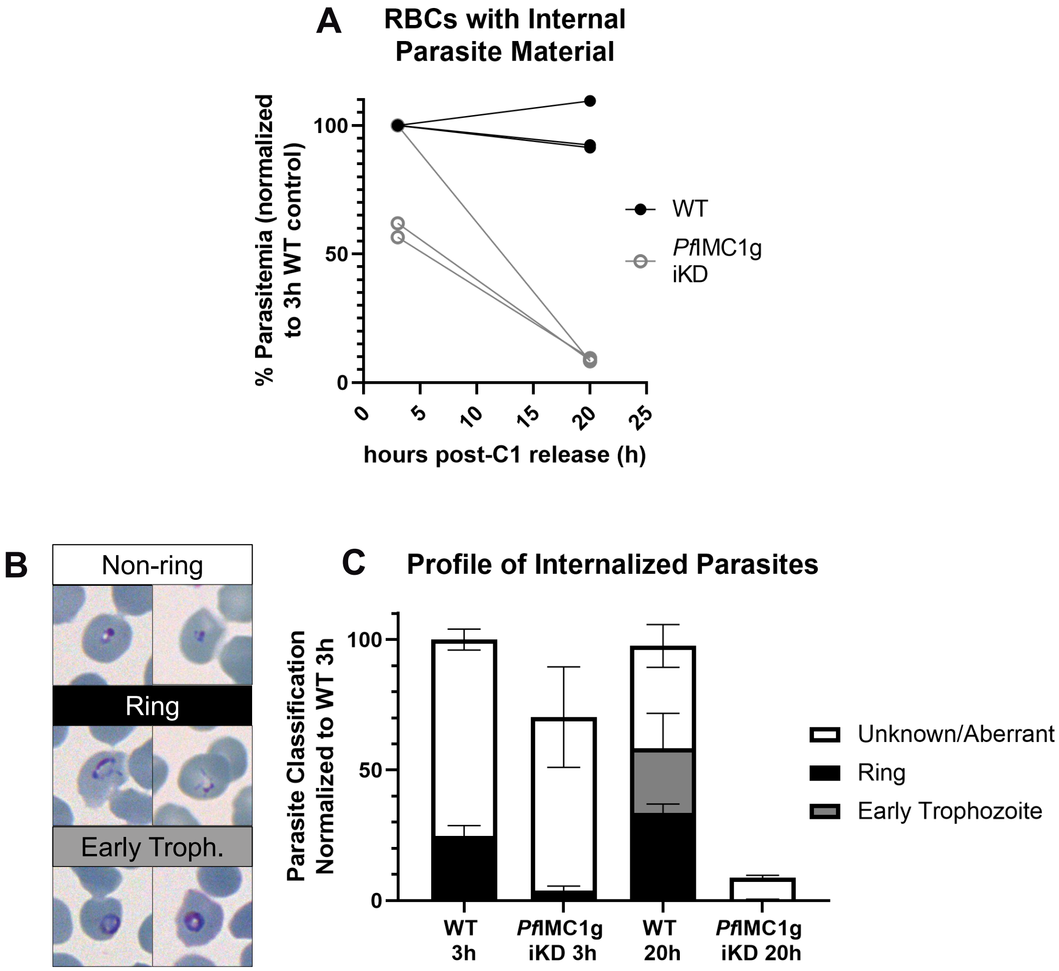

Supplemental Figure 4. PfIMC1g-deficient parasites do not survive internalization. A) Percentage of RBCs staining positive for parasite material by Field's stain (parasitemia) at 3h and 20h. Each biological replicate was normalized to set its 3h WT control to a value of 100. Lines connect samples within the same biological replicate. **B)** Representative images of Field's-stained RBCs positive for parasite material. **C)** Classification of parasite material in RBCs from panel B. Bar height represents parasitemia, normalized to the 3hpi time point of WT parasites as in panel A. Graph shows mean ± SD of 3 biological replicates. h, hours post-C1 release.

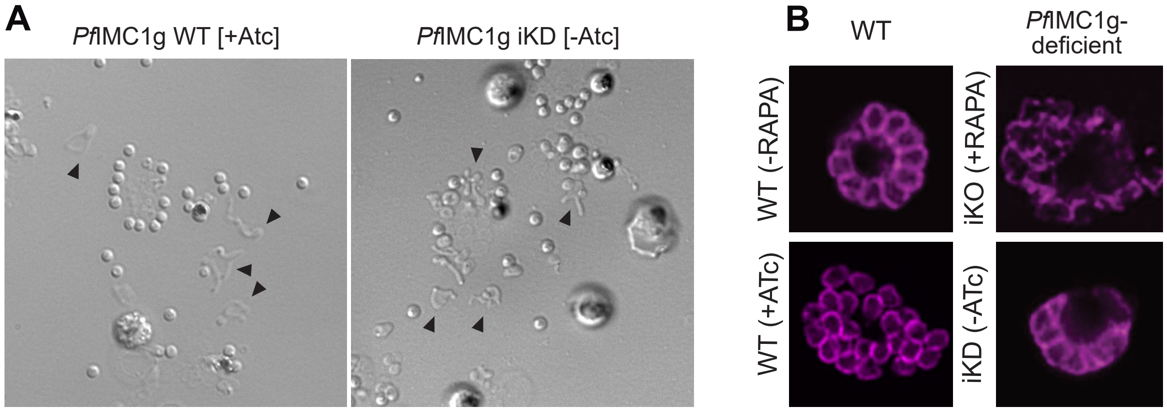

Supplemental Figure 5. *Pf*IMC1g-deficient parasites show signs of compromised structural integrity. A) DIC pictures showing merozoites 1.5h post-egress. Black arrowheads indicate some examples of parasites which have attained amoeboid form. **B)** Slide-based IFAs against IMC marker *Pf*GAP45.

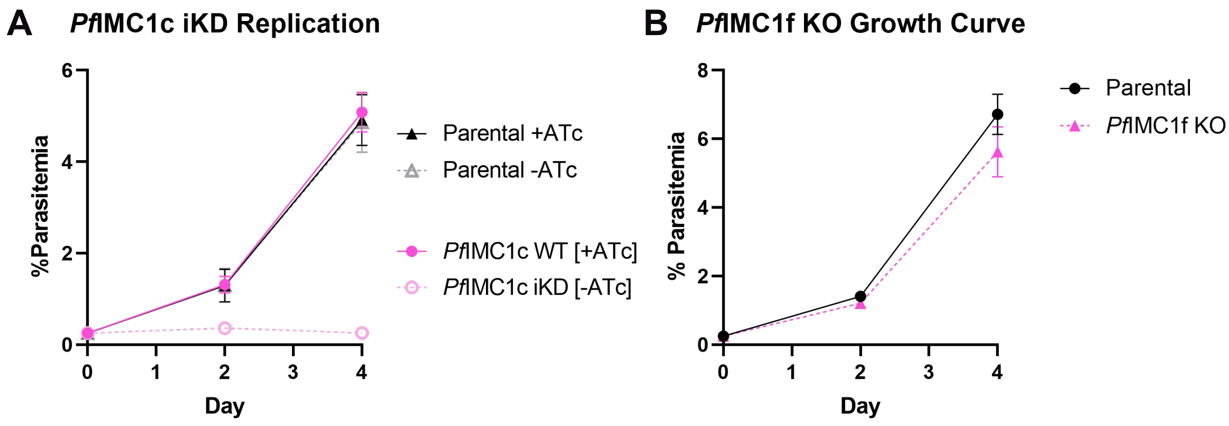

Supplemental Figure 6. *Pf*IMC1c is essential while *Pf*IMC1f is dispensible. A) Replication of *Pf*IMC1c-GGGGSx2-HA WT (+ATc) and KD (-ATc) parasites, in pink circles, and their parental line, in black triangles. **B)** Replication of *Pf*IMC1f KO, in pink triangles, and their parental line, in black circles, as measured by flow cytometry. Graphs show mean ± SD of 3 biological replicates.

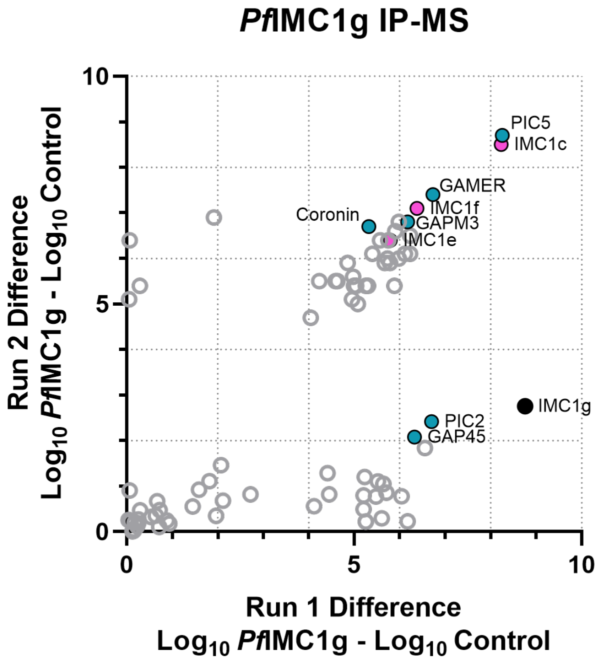

Gene ID	Description	Run 1 Difference	Run 2 Difference	Average coverage
PF3D7_1310700	PIC5	8.26	8.7	19.60%
PF3D7_1003600	IMC1c	8.23	8.5	39.50%
PF3D7_0805200	GAMER	6.734	7.4	12.90%
PF3D7_1351700	IMC1f	6.38	7.1	17.80%
PF3D7_1406800	GAPM3	6.18	6.8	4.90%
PF3D7_1409400	conserved protein, unknown function	5.98	6.8	13.15%
PF3D7_0806800	V-type proton ATPase subunit a, putative	6.23	6.5	10.30%
PF3D7_1436200	BCP1	5.90	6.6	7.70%
PF3D7_0304100	IMC1e	5.80	6.4	12.10%
PF3D7_0109000	PhIL1	6.56	1.84	17.85%
PF3D7_0217800	40S ribosomal protein S26	0.066	6.4	11.2%
PF3D7_0316600	Formate-nitrite transporter	5.57	6.4	2.90%
PF3D7_0515700	GAP 40	1.92	6.9	10.7%
PF3D7_0525800	IMC1g	8.76	2.75	42.50%
PF3D7_0710600	60S ribosomal protein L34	-0.33	6.7	10%
PF3D7_0822900	PIC2	6.70	2.42	15.05%
PF3D7_1104400	Thioredoxin-like mero protein	-0.10	6.4	8.95%
PF3D7_1251200	Coronin	5.32	6.7	10.05%
PF3D7_1347200	nucleoside transporter 1	5.75	6.4	5.95%
PF3D7_1460300	60S ribosomal protein L29, putative	-0.82	7.6	19.45%

Supplemental Figure 7. Enriched proteins identified by IP-MS of *Pf*IMC1g. A) Scatterplot showing proteins with a log10(*Pf*IMC1g/control) ≥ 0 in both biological replicates. Magenta, Alveolins; Blue, known and predicted IMC proteins with log10(*Pf*IMC1g/control) ≥ 2 in both biological replicates. **B)** Top 20 hits from IP-MS (hits with log10(*Pf*IMC1g/control) ≥ 6 .4 in at least one replicate).

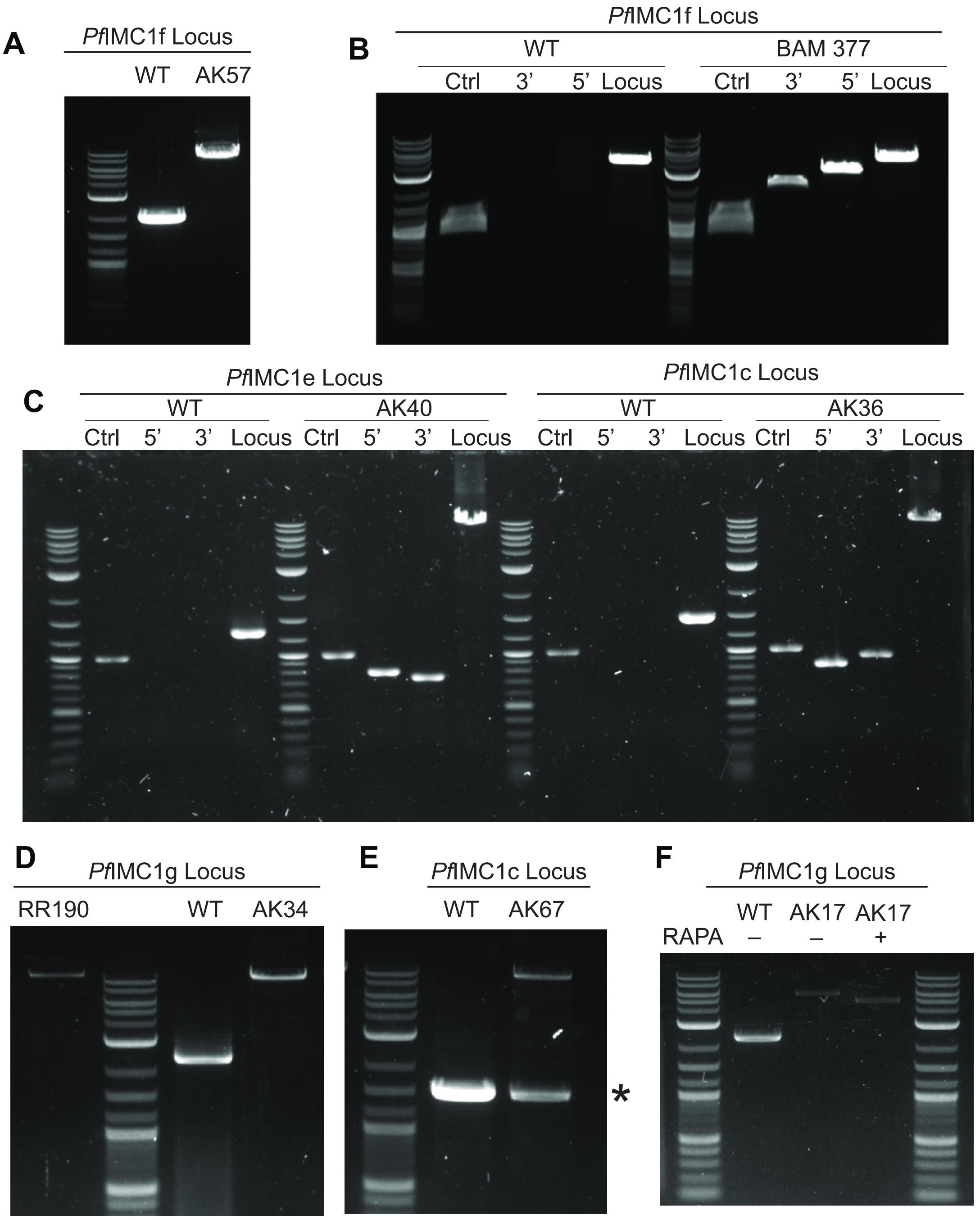

Supplemental Figure 8. Integration check PCRs. A) *Pf*IMC1f-smV5 line, HDR plasmid pAK57. **B)** *Pf*IMC1f KO line, HDR plasmid pBAM377. **C)** *Pf*IMC1e-HA line, HDR plasmid pAK40; *Pf*IMC1c-HA line, HDR plasmid pAK36. **D)** *Pf*IMC1g-smV5 iKD line, HDR plasmid pRR190; *Pf*IMC1g iKD line, HDR plasmid pAK34. **E)** *Pf*IMC1c-GGGGSx2-HA line, HDR plasmid pAK67; * marks the WT locus, indicates the HDR plasmid remained episomal in some parasites and integrated in others. **F)** *Pf*IMC1g-HA iKO line, HDR plasmid pAK17. Excision of *Pf*IMC1g is visible in + RAPA condition. HDR, Homologous Directed Repair; WT, Parental locus PCR; Ctrl, Control PCR; 5’, 5’ Integration Check; 3’, 3’ Integration Check; RAPA, Rapamycin.
