# Supplementary material for: Essential function of alveolin PfIMC1g in the Plasmodium falciparum asexual blood stage
Source: mBio. 2023 Sep 15;14(5):e01507-23. doi: 10.1128/mbio.01507-23 (PMC10653860; doi:10.1128/mbio.01507-23)
Supplement: Video Legend — Legend for Video S1. [file mbio.01507-23-s0002.docx]

**Supplemental Video 1. *Pf*IMC1g-deficient parasites do not form amoeboid rings after internalization.** Time-lapse DIC imaging of RBCs positive for parasite material ~3h post invasion. WT [+ATc] parasite (shown first) shows an amoeboid ring. IMC1g-deficient [-ATc] parasite (shown second) has invaded but was unable to attain amoeboid form.
